# Biotic and environmental stress induces nitration and changes in structure and function of the sea urchin major yolk protein toposome

**DOI:** 10.1038/s41598-018-22861-1

**Published:** 2018-03-15

**Authors:** Immacolata Castellano, Oriana Migliaccio, Giarita Ferraro, Elisa Maffioli, Daniela Marasco, Antonello Merlino, Adriana Zingone, Gabriella Tedeschi, Anna Palumbo

**Affiliations:** 1 Department of Biology and Evolution of Marine Organisms, Stazione Zoologica Anton Dohrn, Naples, Italy; 2 Department of Chemical Sciences, University of Naples Federico II, Naples, Italy; 3 D.I.P.A.V.-Section of Biochemistry, University of Milan, Milan, Italy; 4 Department of Pharmacy, CIRPEB: Centro Interuniversitario di Ricerca sui Peptidi Bioattivi-University of Naples Federico II, Naples, Italy; 5 Department of Integrative Marine Ecology, Stazione Zoologica Anton Dohrn, Napoli, Italy

## Abstract

The major yolk protein toposome plays crucial roles during gametogenesis and development of sea urchins. We previously found that nitration of toposome increases in the gonads of a *Paracentrotus lividus* population living in a marine protected area affected by toxic blooms of *Ostreospsis* cf. *ovata*, compared to control populations. This modification is associated with ovatoxin accumulation, high levels of nitric oxide in the gonads, and a remarkable impairment of progeny development. However, nothing is known about the environmental-mediated-regulation of the structure and biological function of toposome. Here, we characterize through wide-ranging biochemical and structural analyses the nitrated toposome of sea urchins exposed to the bloom, and subsequently detoxified. The increased number of nitrated tyrosines in toposome of sea urchins collected during algal bloom induced structural changes and improvement of the Ca^2+^-binding affinity of the protein. After 3 months’ detoxification, ovatoxin was undetectable, and the number of nitric oxide-modified tyrosines was reduced. However, the nitration of specific residues was irreversible and occurred also in embryos treated with metals, used as a proxy of environmental pollutants. The structural and functional changes of toposome caused by nitration under adverse environmental conditions may be related to the defective development of sea urchins’ progeny.

## Introduction

The ability of organisms to adapt to changing environmental conditions depends, among other factors, on the life history of their parents and on the environmental factors they have experienced in their early life stages. Indeed, environmental factors can either positively or negatively influence the reproductive fitness of parents and this can determine if the offspring lives or dies, especially if it develops in a stressful environment^1^. This phenomenon of non-genetic inheritance is often referred to as trans-generational plasticity and can involve the transfer between generations of processes such as hormonal changes, nutritional provisioning or epigenetics^2^.

Most marine organisms, including echinoderms, are broadcast spawners lacking parental care and releasing their gametes into the seawater for external fertilization^3^. Maternal provisioning is critical for the survival of the offspring of such organisms, especially to guarantee sufficient energy reserves to sustain embryos/larvae until they reach an autonomous developmental stage for external food supply^4^. Because of their sensitivity to the surrounding environment, planktonic embryos and larvae have been extensively used as model organisms in ecotoxicological studies^5–8^. Sea urchins are ubiquitous in the marine benthic environment, where they graze on macroalgal assemblages, and their associated epiphytes, and act as keystone species in confined ecosystems. Their life cycle involves short-lived embryonic and larval stages which metamorphosize into juveniles, and then to mature long-lived adults^3^. The larval stage is a critical phase, as the recruitment success is primarily determined by the survival of the embryos and larvae in the environment they experience^3^. Protective molecular strategies have evolved to allow eggs and early embryos to survive in response to environmental pollutants and marine toxins^9,10^. In the sea urchin *Paracentrotus lividus*, the biosynthetic pathway leading to ovothiol production and the expression of several multidrug efflux genes are regulated by metals and natural algal toxic blooms, thus suggesting a key role of these molecules in the defence mechanism of embryos and larvae from environmental stressors^5–7,9^.

In this scenario, the protein toposome, also commonly referred to as major yolk protein (MYP), plays important roles in the gametogenesis and development of sea urchins, thus deserving special attention. Toposome is first synthesized in the gut of the adult and then secreted into the coelomic fluid of the body cavity, where it is absorbed by the nutritive phagocytes of the gonads in both sexes^11,12^. During gametogenesis, nutritive phagocytes degenerate and toposome is actively endocytosed by the oocytes to form yolk granules^13,14^, which are necessary for the synthesis of proteins and other eggs components. In the eggs, the toposome concentration is maximum before gametogenesis (about 80% of total protein), whereas it decreases with maturation and increases again at the end of the gametogenic cycle^15,16^. Conversely, most toposome is consumed during spermatogenesis^14^, suggesting a key role of the protein for the eggs and not for spermatozoa survival. In *Tripneustes gratilla*, toposome was isolated from membranes or yolk granules as a 22S glycoprotein complex consisting of six identical 160-kDa subunits, each containing several intra-chain disulfide bonds, with a molecular mass of about 900 kDa^17–19^. The 22S glycoprotein complex was identified in the cytoplasm of other sea urchins, including *Paracentrotus lividus*, *Arbacia lixula*, *Lytechinus variegatus*, and *Strongylocentrotus purpuratus*. After fertilization and during early development, mature toposome is proteolytically cleaved in several polypeptides^19^. However, the hexameric structure of the protein is reported to remain intact^19^. Other authors have reported that MYP exists in the ovary of the red sea urchin *Pseudocentrotus depressus* as a tetramer consisting of two disulfide-bonded dimeric subunits with an estimated molecular mass of 600–700 kDa^20–22^.

Different roles have been proposed for toposome in sea urchins. Unuma *et al*. suggested that, the protein plays a nutritional role before spawning both for oogenesis and spermatogenesis, and later for larvae^23,24^. Moreover, in both larvae and adults, toposome is reported to transport zinc which is essential for gametogenesis and the development of various tissues^22,24,25^. During development, toposome was reported to mediate cell adhesion and express positional information^18,19,26–29^. Toposome appeared involved also in restoring structural integrity of damaged plasma membranes of sea urchin eggs or embryonic cells^30^. These biological functions are consistent with the localization of modified toposome from the yolk granules at the level of plasma membranes of all newly formed cells in the embryos^31^.

From the structural point of view, toposome may be considered as a modified iron-less Ca^2+^ binding transferrin, evolutionarily adapted to development and adhesive functions in sea urchin embryos^19^. Although, the importance of toposome for sea urchin development seems to be well accepted, its biological functions remain a matter of debate. Indeed, no clear correlations between Ca^2+^ binding and toposome function have been found, nor any effect of environmental conditions on its structure. Only recently, the influence of external factors on toposome has been observed in *P. lividus* from an area affected by blooms of the toxin-producing dinoflagellate *Ostreopsis* cf. *ovata*. The gonads of the animals were characterized by increased levels of nitric oxide (NO) and a concomitant nitration of toposome^7^. The offspring showed abnormal development associated with increased NO levels and altered expression of NO-regulated genes, thus suggesting that NO accumulated in the gonads can have effects on eggs’ cytoplasmic proteins, thereby transmitted to the embryos.

In this paper, we shed new light on the structure and function relationships of toposome and its post-translational regulation by natural or anthropogenic stressors that sea urchin adults, embryos and larvae can experience in their environment. In order to understand to what extent the post-translational modification affects protein structure and function, toposome from gonads of sea urchins collected at the *O*. cf. *ovata* bloom site and at control site was purified and analyzed in terms of molecular weight, secondary and tertiary structures, and calcium binding properties. The reversibility of toposome nitration and of its effects was investigated in animals detoxified in controlled conditions for different periods. Finally, the nitration of specific residues was compared with that recorded in the toposome of larval stages from sea urchin eggs treated with metals, used as a proxy of environmental pollutants.

## Results

### Toposome nitration in the gonads of *P. lividus* collected at the bloom site and after detoxification

**Figure 1:**
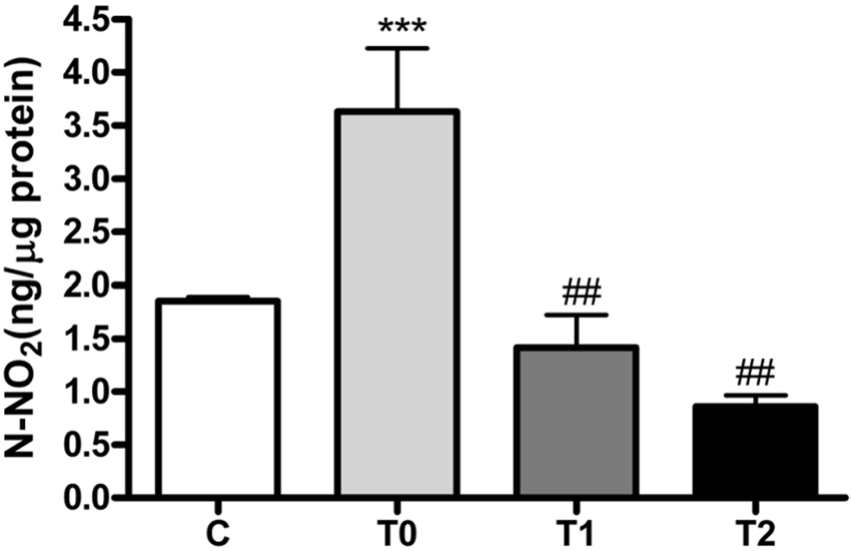
NO levels in the gonads of sea urchins. C, control (pre-bloom animals); T0, animals collected during the toxic bloom; T1, after stabulation for 56 days; T2, after stabulation for 92 days. Data, expressed as ±SD, were assessed by variance analysis (One-way ANOVA, Tukey’s post hoc test). Asterisks represent the significance of the difference compared to the control (***P < 0.001). ^# ^indicates significant difference compared to the T0 (^##^*P* < 0.01) N = 8.

Our previous studies revealed that sea urchins living in an area affected by a toxic bloom of the benthic dinoflagellate *Ostreopsis* cf. *ovata*, the Gaiola Marine Protected Area (MPA) in the Gulf of Naples, were characterized by high levels of NO in the gonads associated with toposome nitration, compared to animals collected at the control site^7^. In order to understand if these processes may be related to the presence of the toxic bloom, sea urchins collected at the bloom phase (T0) in the Gaiola MPA were compared to animals collected prior to the bloom (C) and to animals stabulated in tanks with sea water for 56 (T1) and 92 (T2) days, respectively. Animals collected *in situ* and after stabulation were examined for movement ability, spine losses and feeding behavior, and appeared generally healthy. Ovatoxin-a was found at the concentration of 78.8 µg/kg only in the soft tissue extract of T0 sea urchins, while the toxin was not detected in C animals^7^ and decreased to undetectable levels upon detoxification in T1 and T2 sea urchins. The nitrosative status of the gonads was assessed by measuring endogenous NO levels and NO-induced toposome nitration. NO levels significantly increased in the gonads of T0 compared to C, whereas, after maintenance of the animals in controlled sea water, they decreased in T1 and T2, reaching values comparable with those of specimens collected prior to the bloom (Fig. 1).

**Figure 2:**
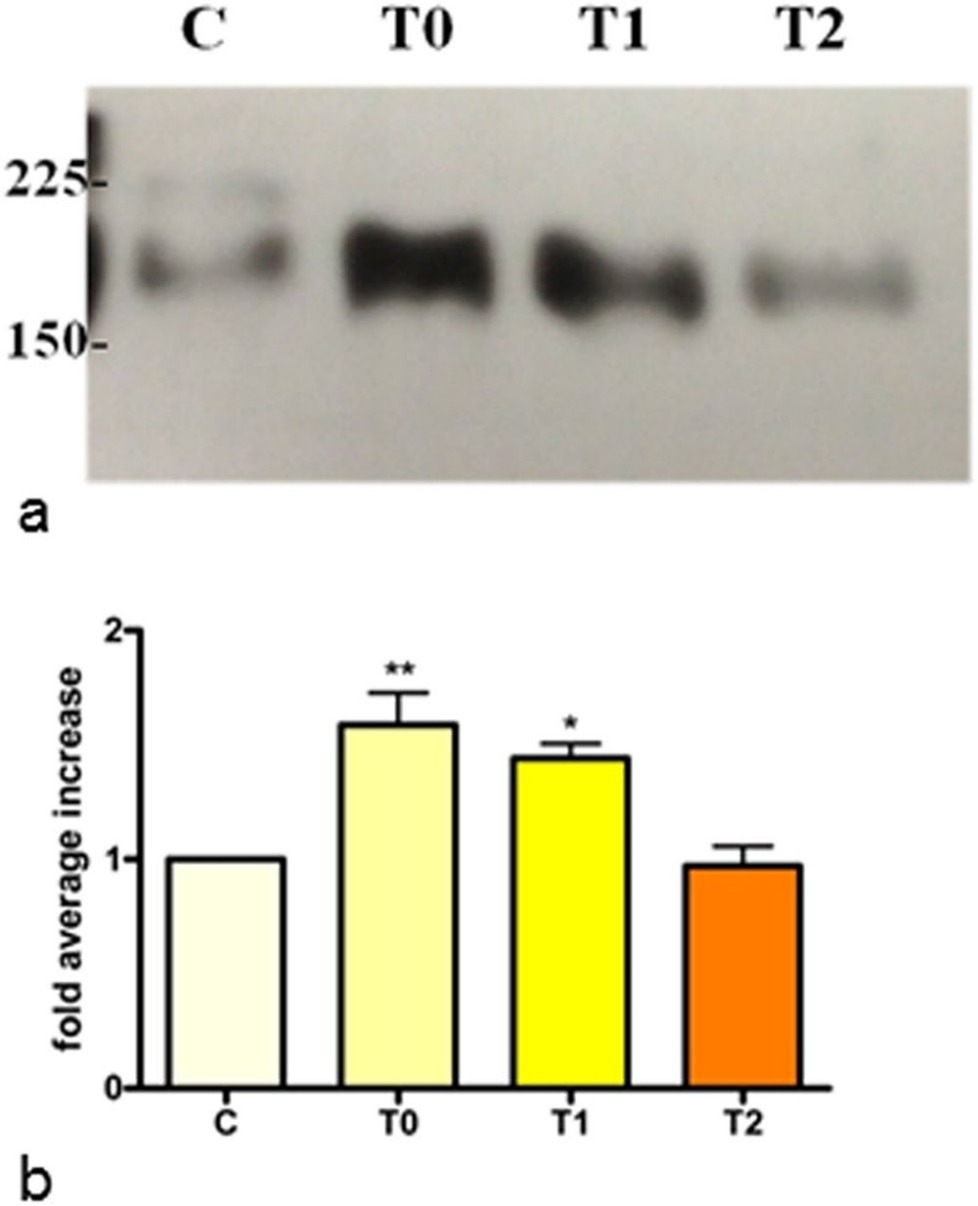
Toposome nitration in the gonads of sea urchins. C, control (pre-bloom animals); T0, animals collected during the bloom; T1, after stabulation for 56 days; T2, after stabulation for 92 days. (**a**) Representative experiment showing the western blot analyzed with anti-nitrotyrosine antibody. (**b**) Histogram showing densitometric analysis of immunopositive toposome respect to control and expressed as fold average increase value ±SE (One-way ANOVA, Dunnet’s post hoc test). The ratio between band intensity values of exposed and control animals was assumed as 1. The bands were quantified by Java Image software. Asterisks indicate significant differences compared to the control (*P < 0.05, **P < 0.01) N = 3.

**Figure 3:**
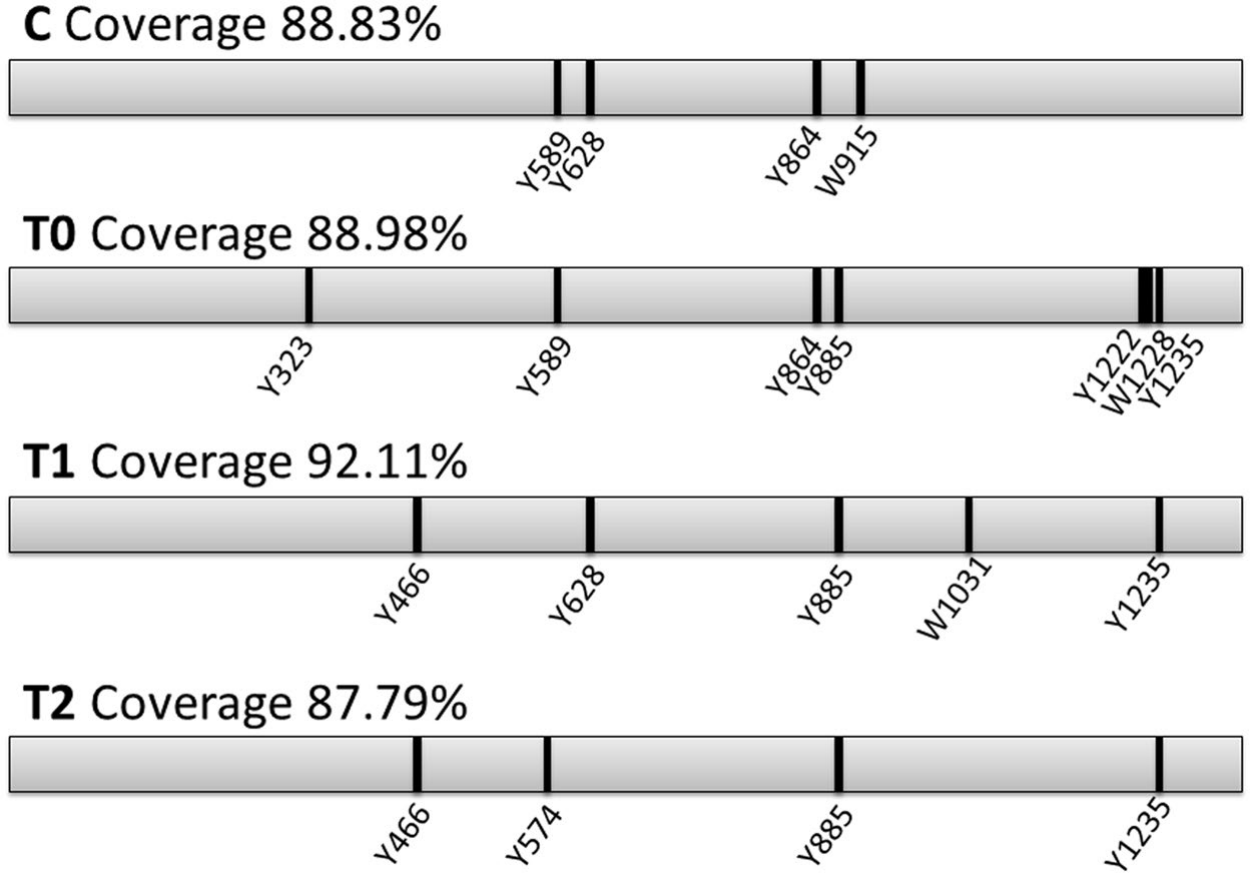
Scheme of nitrated residues of toposome. Sequence coverage and nitrated residues of toposome of sea urchins collected prior to the bloom (C), at the bloom phase (T0), and after stabulation in sea water for 56 (T1) and 92 days (T2). The figure shows the sequence coverage of toposome and the nitrated residues indicated as a black line.

Sea urchin ovary extracts, analyzed by western blot using an anti-nitrotyrosine antibody, showed the presence of the nitrated protein (Fig. 2), previously identified as toposome in T0 samples by tandem mass spectrometry^7^. Densitometric analysis of the immunopositive bands in the extracts revealed a relative increase of nitrated toposome of 1.46 and 1.30 fold in T0 and T1 samples, respectively, compared to C. In T2 sample the level of nitrated toposome decreased becoming comparable to that of the control. To identify the nitrated peptides of the toposome, mass spectrometry analysis was performed on C, T0, T1 and T2 samples. The MS/MS analysis of each trypsin digested sample unequivocally identified toposome (AAQ17121, *Paracentrotus lividus*), also referred to as MYP, as the most abundant protein, with good sequence coverage 88–92% (Fig. 3). Two biological and two technical replicates were run for each sample allowing a robust analysis of the nitrated peptide (Supplementary Table 1). Consistent with the western blot analysis (Fig. 2) the number of nitrotyrosine residues increased during the bloom (T0: 6 nitroTyr) and decreased after stabulation (T1: 4 nitroTyr, T2: 4 nitroTyr) compared to the control (C: 3 nitroTyr). The number of the identified nitrated tryptophans did not change in T0 and T1 samples compared to control. The nitrated peptides reported in Supplementary Table 1 are also illustrated in a cartoon of the toposome sequence (Fig. 3). The residues that we found nitrated under these conditions are well conserved in the protein from different species and are located in the central and in the C-terminal domain of the toposome, while the N-terminal is not modified (Supplementary Figure 1). In particular, the residues Y885 and Y1235, found nitrated in T0 and not in C, remain modified also after stabulation in T1 and T2.

### Effect of tyrosine nitration on the toposome structure

It is well known that incorporation of a nitro group into protein tyrosines can lead to significant structural and functional modifications^32^, therefore, the different number of nitrated tyrosines observed in T0 compared to C animals, could be associated with conformational variations induced by the oxidative post-translational modification. In order to verify the effect of the tyrosine nitration on the structure of toposome, the protein was purified from *P. lividus* gonads and analyzed in terms of molecular weight by gel filtration chromatography. Toposome from C eluted as a protein complex with an apparent molecular weight of 517 kDa, whereas T0 eluted as a protein complex of 446 kDa.

**Figure 4:**
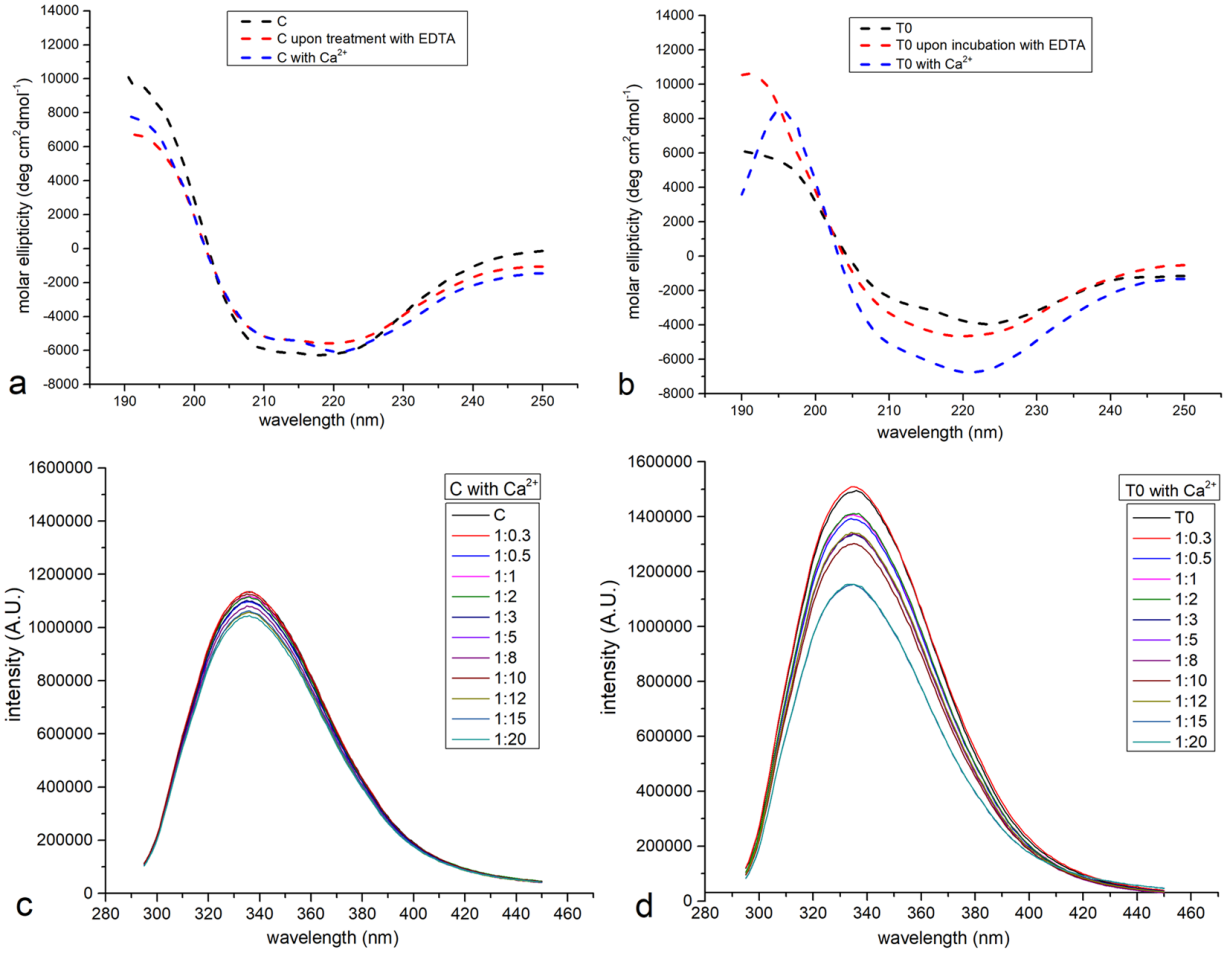
Structural properties and Ca^2+^ affinity of toposome. Far UV CD and intrinsic fluorescence spectra of toposome purified from control and T0 animals. CD spectra (panel a,b) measured in 10 mM Tris-HCl buffer at pH = 7.8 and 10 °C. Spectra of the proteins (0.1 mg mL^−1^) were compared with that obtained for a sample upon treatment with EDTA and after addition of 1 mM Ca^2+^. Intrinsic fluorescence spectra (panel c,d) measured in 10 mM Tris-HCl buffer at pH = 7.8 and 20 °C. Spectra of the proteins (0.05 mg mL^−1^) were compared with those obtained for the protein upon addition of increasing concentration of Ca^2+^ (protein to Ca^2+^ molar ratio between 1:0.3 and 1:20).

The spectroscopic features of C and T0 toposome were compared through far-UV circular dichroism (CD) (Fig. 4a,b) and fluorescence spectroscopy (Fig. 4c,d) analyses, at 10 °C and 20 °C, respectively. CD spectrum of C (Fig. 4a) was characterized by the presence of two negative minima at 208 and 222 nm and a positive signal at 190–200 nm which are typical fingerprints of proteins that possess a folded conformation in solution with a mixed alpha/beta structure. The CD spectrum of T0 was similar (Fig. 4b), but minima at 208 and 222 nm were slightly shallower, thus indicating a lower helical content in the protein with a higher number of nitrated tyrosines. These findings indicate that nitration has a small, but significant effect on the secondary structure of the protein. Indeed, deconvolution of the spectra reveals that T0 protein possesses 35–40% of alpha helices and about 20% of beta strands, whereas C protein has 40–45% of alpha helices and 20–25% of beta strands.

Intrinsic fluorescence in proteins is mainly due to the presence of tryptophans, because phenylalanines have a very low quantum yield, and emission by tyrosines is often quenched. Tryptophan residues were selectively excited at 295 nm, because at this wavelength there is no absorption by tyrosine, but it is not possible to selectively excite tyrosines. The intrinsic fluorescence emission spectra of C and T0 (Fig. 4c,d and Supplementary Figure 2a,b) shows that there are no shifts in λ_max_, which corresponds to 337 nm in both C and T0, indicating that tryptophan residues are similarly buried in the hydrophobic core in the differently nitrated proteins.

### Effects of tyrosine nitration on toposome Ca^2+^ binding properties

In order to investigate the effects of tyrosine nitration on the reported ability of toposome to bind calcium ions, we studied the effect of Ca^2+^ binding to C and T0 structures by using CD and fluorescence emission. In order to perform a direct comparison between the apo form and the Ca^2+^-bound form of the protein, purified C and T0 were subjected to a pre-treatment with EDTA (see Methods). Interestingly, CD spectra of the proteins after this treatment slightly change when compared to those of the purified untreated proteins (Fig. 4a,b; see also Supplementary Figure 2c,d). This result suggests the release of bivalent or trivalent ions from the protein upon EDTA treatment. Subsequently, we verified that the protein from *P. lividus* actually binds Ca^2+^ by registering fluorescence spectra of the protein at increasing concentrations of the ion and estimated the affinity of the protein for this bivalent ion by isothermal titration calorimetry (ITC). Fluorescence spectra of C and T0 at different protein to metal ion ratios are reported in Fig. 4c,d. Upon addition of Ca^2+^, in both cases, a small but significant decrease of fluorescence intensity was observed (quenching). T0 experienced a more pronounced quenching at increasing concentrations of Ca^2+^ when compared to C, suggesting a higher affinity for Ca^2+^ of the protein with a higher number of nitrated residues.

**Figure 5:**
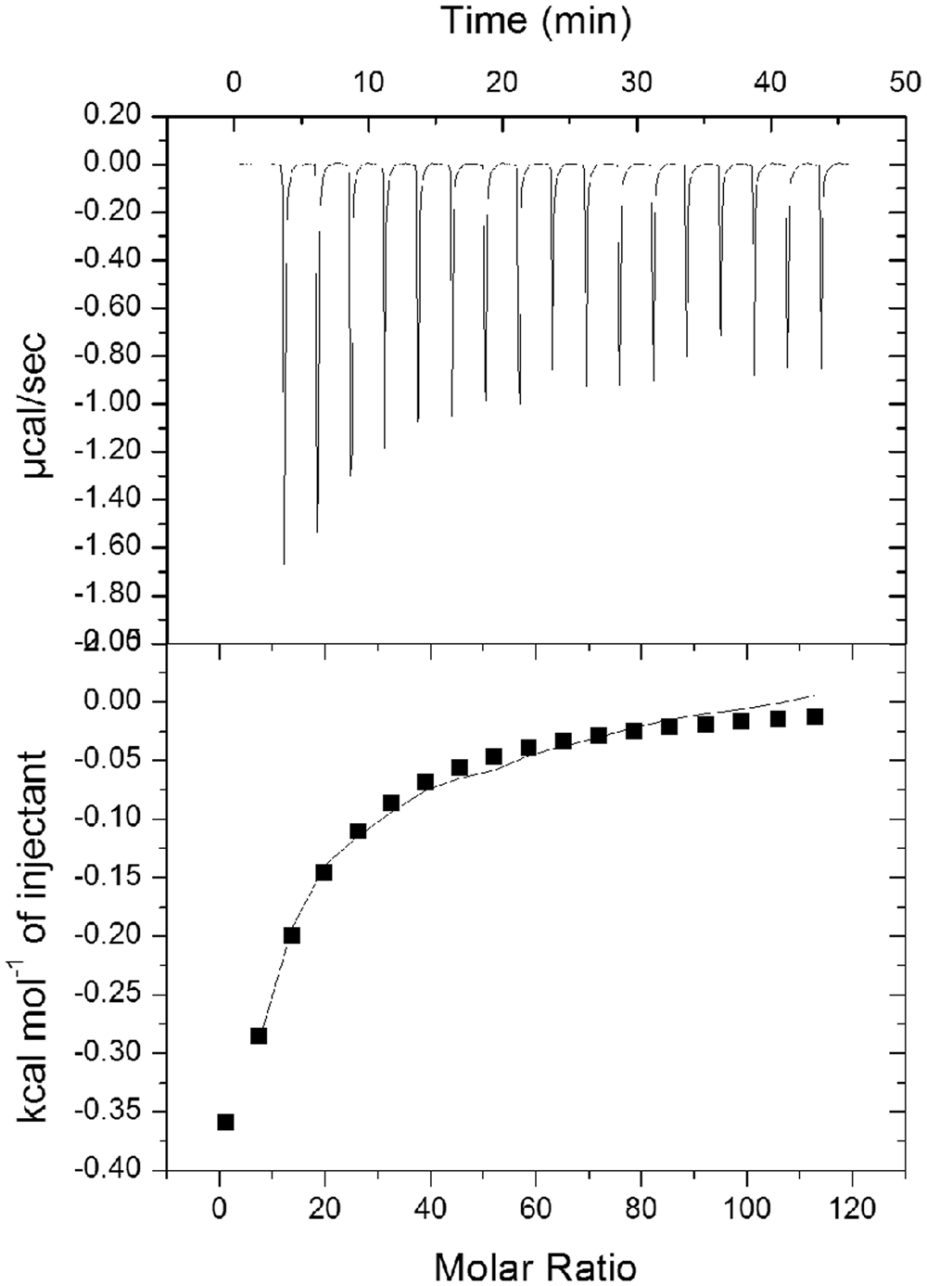
Ca^2+^ binding properties of toposome. ITC Calorimetric raw and integrated data for C (20 µM) titration with CaCl_2_ (2 mM). Data fitting was achieved with ‘one set of sites’ model.

ITC data confirmed Ca^2+^ binding to C. In particular, by titrating aliquots of CaCl_2_ into protein solution, the downward ITC titration peaks demonstrated that the association between Ca^2+^ and the protein is an exothermic reaction, as shown in Fig. 5. Data reached saturation and allowed an estimation of the thermodynamic parameters: the entropic value (ΔS) was 2.67 cal/mol/deg and the binding affinity constant (Ka) 1.4 ± 0.2 × 10^3^ M^−1^, while the enthalpic contribution was quite low (ΔH = −3.5 ± 0.8 kcal mol^−1^). Unfortunately, the affinity of T0 for Ca^2+^ was not estimated under the same experimental conditions, because the protein precipitated in the effort to reach 20 µM concentration.

Furthermore, we evaluated the changes of CD spectra induced by the presence of Ca^2+^. Ca^2+^ binding to EDTA-treated samples induces significant structural modifications, as shown by the comparison of the spectra reported in Fig. 4a,b. These variations are more marked in the case of T0, consistently with the higher quenching of fluorescence intensity observed in the titration with Ca^2+^ of this protein when compared to the control. The conformational stability of T0 and C and that of the Ca^2+^ bound forms were also studied by thermal unfolding CD experiments (Supplementary Figure 3). Denaturation curves of both C and T0 present a sigmoidal curve with a single inflection point, corresponding to T_m_ value of 52–53 °C, both in the absence and in the presence of Ca^2+^. Thermal unfolding proved to be a reversible process: renaturation of C and T0 upon decreasing temperature shows a full recovery of the spectral features of the proteins. These findings indicate that the protein unfolds according to a reversible two-state N ⇔ D model (N = native, D = denatured), with the presence of Ca^2+^ that does not significantly affect protein overall thermal stability.

### Cleaved nitrated toposome in developing embryos exposed to cadmium and manganese ions

**Figure 6:**
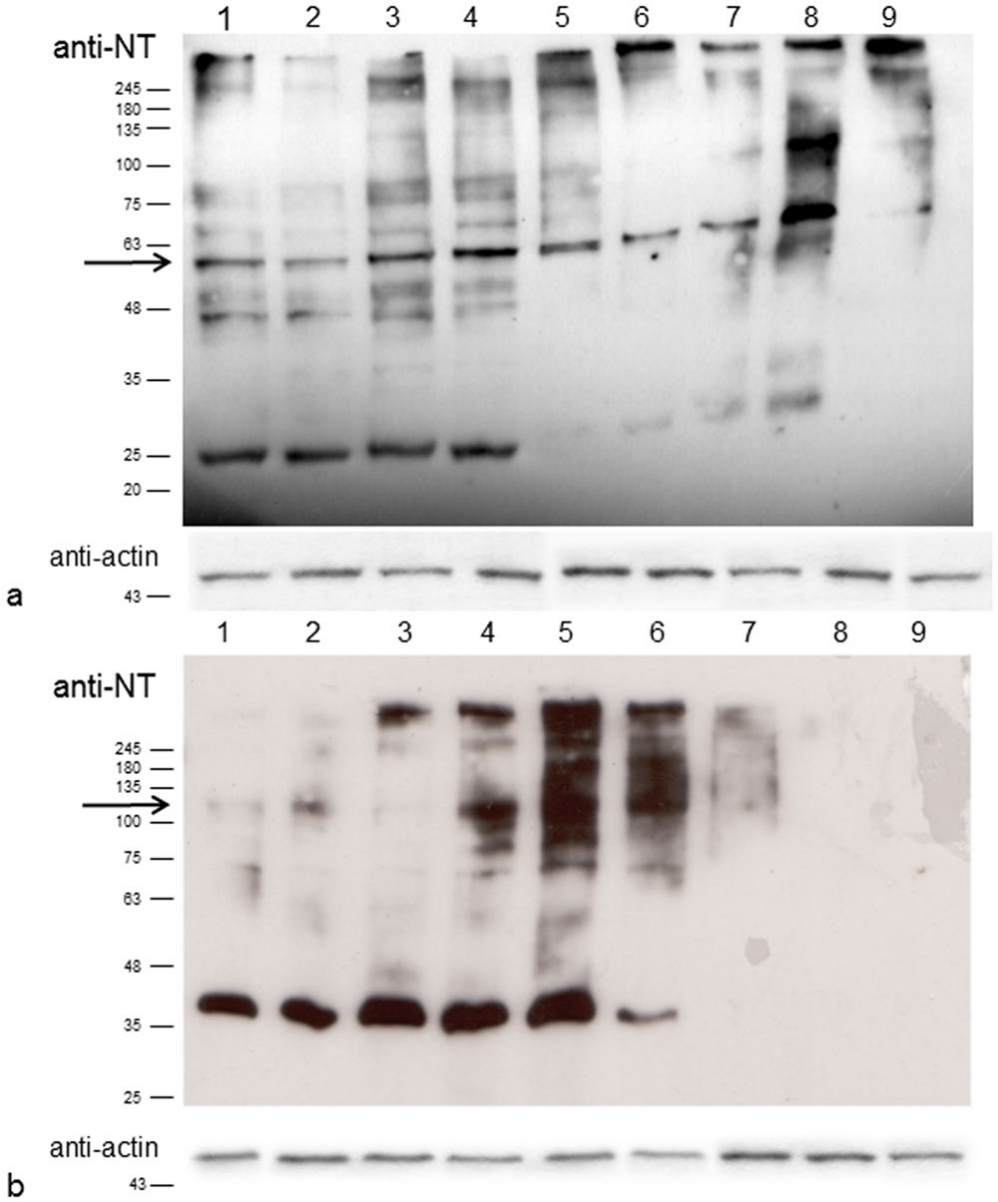
Pattern of nitrated proteins in sea urchin embryos after metals treatment. (**a**) Early blastula stage. (**b**) Swimming blastula stage. 1 = Control in sea water, 2 = Mn^2+^ 1.8 × 10^−5^ M, 3 = Mn^2+^ 3.6 × 10^−5^ M, 4 = Mn^2+^ 7.8 × 10^−5^ M, 5 = Mn^2+^ 15.5 × 10^−5^ M, 6 = Mn^2+^ 31.2 × 10^−5^ M, 7 = Cd^2+^ 5 × 10^−7^ M, 8 = Cd^2+^ 10^−6^ M, 9 = 5.2 × 10^−6^ M. Representative experiment showing the western blot analysis with anti-nitrotyrosine (anti-NT) antibodies. Anti-actin is used as loading control.

To investigate if NO can induce also the nitration of polypeptides arising from toposome proteolysis during sea urchin development, we have followed by western blot analysis the formation of tyrosine-nitrated proteins in developing embryos exposed to different concentrations of cadmium and manganese ions compared to untreated embryos. No protein nitration was found during the early developmental stages, 2-cell and 8-cell stages. The major changes occurred at early blastula and swimming blastula stages. In particular, at early blastula stage, a nitrated band at 60 KDa was evident at Mn^2+^ concentrations of 3.6 × 10^−5^ M and 7.8 × 10^−5^ M, compared to control embryos in sea water (Fig. 6a). The same nitrated band was evidently increased following exposure to Cd^2+^ treatment at 10^−6^ M. Moreover, a nitrated band at 120 kDa was observed at swimming blastula stage when Mn^2+^ concentrations from 7.8 to 31.2 × 10^−5^ M were used (Fig. 6b). No changes were observed during the prism stage, whereas an increase in the protein nitration at pluteus stage was recorded only at high Mn^2+^ concentration.

**Figure 7:**
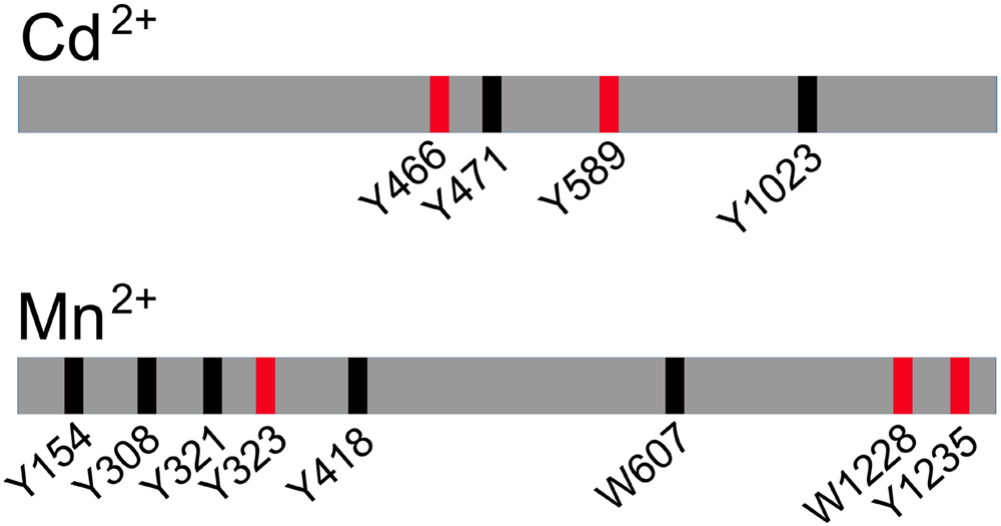
Scheme of nitrated residues of toposome after metals treatment. Nitrated residues are indicated as a black line. In red the residues found nitrated also in samples collected at bloom (T0) or after stabulation (T1, T2) in sea water (see Fig. 3 for the comparison).

The nitrated bands at 60 kDa from early blastula stages after treatment with Cd^2+^ (10^−6^ M) and or Mn^2+^ (7.8 × 10^−5^ M) as well as the band at 120 kDa from swimming blastula stages upon treatment with Mn^2+^ (7.8 × 10^−5^ M) were both identified as toposome by mass spectrometry analysis (Supplementary Table 2). The analysis of the nitrated bands allowed also the identification of the peptides nitrated at tyrosine and tryptophan level after Cd^2+^ and Mn^2+^ treatments (Fig. 7; Supplementary Figure 1). Among the nitrated tyrosine residues, some corresponded to those identified in toposome from the animals collected at T0 and after stabulation at T1 and T2 (see Fig. 3). In details, with Cd^2+^, the nitrated Y466 was also present in T1 and T2 samples and Y589 occurred also in T0 sample. With Mn^2+^ Y1235 was also present in T0, T1 and T2, whereas Y323 and also W1228 were present only in T0 samples.

## Discussion

Considering the key role that toposome plays during gametogenesis and embryo development, its regulation by environmental factors assumes crucial importance in the health, fate, and fingerprint of future generations. The results of this study provide evidence that NO, most likely produced as a consequence of stressful conditions, induces nitration of toposome in the gonads of sea urchin adults as well as in its cleaved form during development. The mechanism underlying NO levels increase are unknown. Based on previous evidence of a nitric oxide synthase (NOS) gene regulation during development of *P. lividus* exposed to the toxic bloom^7^, a similar NOS gene regulation in sea urchin gonads can be hypothesized. However, we cannot exclude a possible regulation of NOS enzyme by Ca^2+^, whose levels can change upon environmental stress^33^. Moreover, this study reports that nitration of the gonadic toposome results in a slight effect on the protein structure and an increase of the Ca^2+^ binding properties, which may affect toposome biological functions.

Sea urchins living in an area affected by *Ostreopsis* cf. *ovata* blooms and grazing on macroalgae epiphytized by this dinoflagellate accumulate ovatoxin in their internal organs and exhibit a greater amount of nitrated tyrosine residues in toposome of gonads compared to sea urchins harvested prior to the bloom phase^7^. Although other possible impacts of the bloom forming species cannot be excluded, it is likely that the nitrosative stress in the gonads, resulting in the increase of toposome nitration, is induced by the ovatoxin accumulated in the sea urchin tissues, after ingestion. Since the sequence of toposome contains 50 Tyr residues, the nitration of 5–6 tyrosines may be considered as a selective process, by which the local environment of tyrosine residues drives the reaction with NO and governs its selectivity^34^. Interestingly, the number of nitrated residues increases in toposome from gonads of animals that have accumulated ovatoxin, whereas it decreases in gonads of sea urchins stabulated under controlled conditions, supporting the hypothesis of the toxin as the causative agent of the NO levels increase and of the consequent toposome nitration. However, despite the undetectable toxin amounts after 3 months of stabulation, some post-translational modifications persist over the time, indicating that the effects of the stressful conditions are partially irreversible, as in the case of the two tyrosines, Y885 and Y1235, which remain nitrated also after sea urchin stabulation. Hence, the toposome modifications occurring during the bloom can have implications in the following months, when the reproductive season of sea urchin begins, and fertilization of the eggs containing nitrated toposome can imply the transgenerational transmission of the NO-regulated processes^7^.

Most of the same tyrosine residues, nitrated in the toposome after exposure of sea urchins to marine toxic bloom, were also target of nitration during embryo development upon exposure to other stressors, e.g. metals in environmentally relevant concentrations, thus suggesting that NO actually drives specific post-translational modifications. This finding also indicates that some residues remain exposed on the protein surface for targeting by nitration also after protein cleavage (see the residue Y1235, for example), occurring in the embryos. In these conditions, the increased tyrosine nitration of toposome can be related to the anomalies previously found in embryos and larvae treated with metals at the same concentrations^5^. The differences in polypeptides derived from toposome at different embryonic stages^19^, as well as the differences in the level of nitration in some stages compared to the others, may be ascribed to cellular and tissues remodeling during development, which also implies protein degradation and new protein synthesis.

Toposome from the eggs of the sea urchin *Strongylocentrotus droebachiensis* has been recently characterized as a protein of 240 kDa, which, under reducing conditions, gives a band at 170 kDa^35^. Our finding that toposome from animals collected at pre-bloom phase (C) and at bloom phase (T0) eluted as protein complexes of 517 and 446 kDa, respectively, indicates that both proteins behave as oligomers formed by three subunits. However, the slightly different mobility of the two proteins suggests that toposome from T0 animals has a more compact structure and reduced flexibility than in C animals. This property could be linked to slight differences observed in the secondary structure content, and to altered solubility of toposome from T0, which could account for the proneness of the protein to aggregate and precipitate in the presence of Ca^2+^. Indeed, protein solubility depends on the distribution of hydrophilic and hydrophobic residues on its surface, and nitration can alter the protein surface charge, thus significantly affecting protein solubility. As a consequence, nitration can induce protein aggregation and precipitation^36^.

Moreover, the small enthalpic contribution (ΔH = −3.5 ± 0.8 kcal mol^−1^) of the Ca^2+^ interaction confirms the idea that the protein-metal ion recognition process occurs on the protein surface, and can be classified as a “surface binding” process (mainly toward carboxylate or nitrated groups of the protein side chains). This can determine small variations of hydrogen bonding network and interaction of the protein with solvent molecules. Therefore, nitrated residues on the surface of the protein can actually affect the binding of Ca^2+^. This hypothesis is confirmed by the finding that, although the affinity of the protein for calcium ions is low in agreement with what has been previously suggested on the basis of indirect binding assays^30^, Ca^2+^ binding to EDTA-treated samples induces more pronounced structural modifications in the case of T0 compared to C. One speculation could be that the higher affinity for Ca^2+^ of the protein with a higher number of nitrated residues (T0) could be linked to the higher number of negative charges that are present on the protein surface upon nitration.

We can finally speculate that increased nitration of toposome and consequent changes in the affinity of protein for Ca^2+^ can alter the buffer capacity of the cytoplasm for the ion, considering the high concentration of toposome in gonadic tissues. In this view, as Ca^2+^ and NO play key roles in reproduction and development of the sea urchins^37–42^, the changes in toposome nitration may affect fundamental molecular pathways driving to anomalies in the embryos. Indeed, in our previous study we have reported that the progeny development of sea urchins exposed to *O. ovata* bloom was negatively affected in terms of gene expression profile and phenotypic anomalies^7^. A further hypothesis could be that the changes in the affinity of Ca^2+^, which is reported to affect the adhesion properties of toposome^18,26,29^, could induce changes in cell embryo membrane-membrane interactions and the consequent developmental process^30,43^.

In conclusion, this study highlights that post-translational modifications induced by environmental factors through NO-mediated mechanisms can affect the function of the toposome in sea urchins. Considering that, maternal provisioning influences the successful development and survival of offspring, thus impacting adult populations, NO-induced modifications of toposome can be considered as an environmentally-driven trait change, likely transmitted to future generations, with consequences at the population and ecosystem levels.

## Methods

### Ethics statement

*Paracentrotus lividus* (Lamarck) sea urchins were collected in the Gulf of Naples at the Gaiola Marine Protected Area at station G1 (40°47.494′N, 14°11.282′E), with the authorization of the Soprintendenza Speciale per i Beni Archeologici di Napoli e Pompei. Animals were also collected in the Gulf of Naples, near Castel dell’Ovo (Lat. 40°49′41″, Lon. 14°14′48″) from a location that is not privately-owned or protected in any way, according to the authorization of Marina Mercantile (DPR 1639/68, 09/19/1980 confirmed by D. Lgs. 9/01/2012 n. 4). The field studies did not involve endangered or protected species. All animal procedures were in compliance with the guidelines of the European Union (directive 2010/63 and following D. Lgs. 4/03/2014 n. 26).

### Sea urchin collection, maintenance and handling

*P. lividus* with mean size of 4.5 ± 1.5 cm in diameter were collected by SCUBA divers at the Marine Protected Area of Gaiola in the Gulf of Naples prior to the *O*. cf. *ovata* bloom (June 2012) and at bloom phase (July 2012). Animals were transported in insulated boxes to the laboratory within 1 h and maintained in tanks with sea water at least for 1 night. For stabulation experiments, sea urchins were kept in tanks with circulating sea water, at 18 ± 2 °C and with 12:12 light: dark cycle. The density of the individuals in the tanks was maximum 1 animal/5 litre. Every day a check of the animals was performed. In particular, macroscopical observations (i.e. spines folded over or lost), feeding ability and movements were analyzed to identify possible negative effects on their health. Every 3 days, animals were fed by using fresh macroalgae (*Ulva lactuca*). After 56 and 92 days, the animals were sacrified. For toxicity tests, the sea urchin internal content was collected. Toxin extraction and determination of ovatoxin-a by LC-MS TOF was performed at the Istituto Zooprofilattico Sperimentale del Mezzogiorno, Portici, Napoli, as previously reported^7^. The gonads from sea urchins were removed, weighed, washed with PBS, frozen in liquid nitrogen and kept at −80 °C until analysis. For experiments with metal ions, sea urchins were collected during the breeding season at Castel dell’Ovo in the Gulf of Naples and maintained as described above.

### Gamete collection and embryo culture

Gamete collection and fertilization were performed as previously described^5^. Fertilized eggs (150 eggs/mL), after 5 min from fertilization, were treated with the metals at the different concentrations^5^. Embryo and larvae cultures (about 30,000 embryos/larvae) were collected by centrifugation (1800 rpm, 10 min at 4 °C) at different developmental stages. The pellets were washed with PBS, frozen in liquid nitrogen and kept at −80 °C until analysis.

### NO determination

Gonads were homogenized in 20 volumes of PBS and centrifuged at 25,000 × g for 20 min at 4 °C. The supernatants were analyzed for NO levels by monitoring nitrite formation with the Griess reaction, as previously described^5,44^. Aliquots (300 µl) of the supernatant were incubated for 2 h, at room temperature (25 °C) with nitrate reductase (1 U/ml) and enzyme co-factors FAD (100 μM) and NADPH (0.6 mM). Samples were incubated for 10 min in the dark with 300 µl of 1% (wt/v) sulphanilamide in 5% H_3_PO_4_ and then for 10 min with 300 µl of 0.1% (wt/v) N-(1-naphthy)-ethylenediaminedihydrochloride. The absorbance at 540 nm was determined and the molar concentration of nitrite in the sample was calculated from a standard curve generated using known concentrations of sodium nitrite (0–100 μM). Each sample was determined in triplicate. The efficacy of nitrate reduction by nitrate reductase was determined on known concentrations of nitrate and nitrite recovery was 90–100% over the entire range of sodium nitrite. The coefficient of variation between the different experiments was less than 5%.

### Protein extraction and immunoblotting

Gonads were homogenized on ice in 20 volumes of 10 mM Tris-HCl buffer at pH 8.0, and 10 mM NaCl, supplemented with 0.1 mM phenylmethylsulfonylfluoride (PMSF), as previously reported^14^. Lysates were centrifuged at 25,000 × g for 20 min at 4 °C. Supernatants were collected and total protein concentration of the extract was determined using a Bio-Rad Protein Assay Reagent (Bio-Rad, Milan, Italy) with bovine serum albumin as a standard. Proteins from different developmental stages were extracted by homogenizing the pellets on ice for 10 minutes in two volumes of RIPA lysis buffer (150 mM NaCl, 50 mM Tris-HCl pH 7.6, 5 mM EDTA, 0.5% NP-40, 0.5% sodium deoxycholate, 0.1% SDS) supplemented with PMSF 1 mM, complete protease inhibitor cocktail (Roche Monza, Italy) and phosphatase inhibitor cocktail (PhosphoSTOP, Roche). Lysates were centrifuged at 10,000 rpm for 20 min at 4 °C. Supernatants were collected, and total protein concentration was determined as described above. Before electrophoresis, protein samples used for Coomassie staining were incubated at 85 °C for 5 min, whereas samples examined for nitrated proteins were not. Equal amounts (10 µg) of protein extracts were separated by SDS-PAGE under reducing conditions on 7.5% gels. Following SDS-PAGE, gels were stained with Coomassie or blotted onto polyvinylidendifluoride (PVDF, Sigma-Aldrich) membrane. To detect nitrated proteins, the membrane was analyzed with monoclonal anti nitrotyrosine antibody (1: 20,000) (Invitrogen). After washing in 20 mM Tris pH 7.6, 137 mM NaCl and 0.1% Tween 20, labeled proteins were detected by Amersham ECL Prime Western Blotting Detection Reagent (GE Healthcare, EuroClone, Milan, Italy).

### Toposome purification and molecular mass determination

Toposome was purified from gonads extracts by gel filtration liquid chromatography. About 5–6 mg of total extract was filtered through a 0.2 μm filter and loaded onto a Sephacryl S-400 HR column (1.8 × 42 cm, GE Healthcare) eluted with Tris-HCl buffer, pH 8.0, containing 150 mM NaCl, at a flow rate of 0.5 ml/min. Size determination was made by comparison with molecular mass standards (Sigma), chromatographed under the same conditions. The molecular mass standards used were as follows: blue dextran 2,000 kDa, thyroglobulin, 669 kDa; apoferritin, 443 kDa.

### Liquid Chromatography Electrospray Tandem Mass Spectrometry (LC–ESI-MS/MS)

For protein identification, after electrophoresis each band was cut and digested *in situ* by trypsin sequence grade upon extraction with TCA (trichloroacetic acid) and acetonitrile (CH_3_CN), reduction with 45 mM dithiothreitol and alkylation with 100 mM iodoacetamide^45^. MS/MS analysis was carried out by a LTQ-Orbitrap Velos (Thermo FisherScientific), as previously described^46^. Data Base searching was performed using the Sequest search engine of Proteome Discoverer 1.4 (Thermo Fisher Scientific). The identification of the peptides with nitrated tyrosine or tryptophan was carried out by Tandem Mass Spectrometry. The data are the results of two different experiments. To increase the confidence of the identification only peptides nitrated in both experiments and with Xcorr at least equal to 1.5 were considered.

### EDTA treatment

To remove calcium or other ions present in the purified samples, C and T0 were incubated with EDTA in a molar ratio of 1:10 protein to EDTA at 4 °C. After 24 hours of incubation, the samples were dialysed overnight against 10 mM Tris-HCl at pH 7.8.

### Isothermal titration calorimetry (ITC) experiments

ITC experiments were performed with an iTC200 calorimeter (Microcal/GE Healthcare), using 2 mM CaCl_2_ in the syringe and 20 µM toposome (C) in the cell, in 10 mM Tris-HCl buffer, pH 7.8. The heat generated per injection as Ca^2+^ bound to the protein was recorded and displayed as differential power (μcal s^−1^) vs. time (min). The area under each injection peak was integrated and presented as kcal mol^−1^ of injectant vs. the molar ratio of [Ca^2+^]/[protein]. Subtracting the integrated peak areas for ligand/buffer titration from the ligand/protein titration allows a direct determination of thermodynamic parameters. Data were fitted to a “one set of sites” binding model with Origin software (GE Healthcare).

### Circular dichroism measurements

Far-UV CD spectra of C and T0 (0.1 mg mL^−1^) were collected in 10 mM Tris-HCl buffer, pH 7.8, at 10 °C using a JASCO J-815 spectropolarimeter equipped with a Peltier block arrangement (PTC-423S/15). A quartz cuvette of 0.1 cm path length was used. Raw ellipticity data were converted to mean residue ellipticity using the formula [θ] = [θraw × 100 × MRW]/c × 1, where MRW is the mean residue weight for C, c is the concentration of the protein in mg mL^−1^ and 0.l is the path length in cm. Thermal denaturation curves of C and T0, in the absence and in the presence of 1 mM Ca^2+^, were constructed by recording the CD signal at 222 nm as a function of temperature using a scan rate of 1 °C min^−1^. Deconvolution of CD spectra for secondary structure amount has been performed using CDNN software^47^.

### Fluorescence measurements

Intrinsic fluorescence emission spectra for C and T0 were collected at 20 °C using a HORIBA Scientific Fluoromax-4 spectrofluorometer upon excitation at λ_exc_ = 280 nm or λ_exc_ = 295 nm. Spectra were registered between 295–310 and 450 nm, using a protein concentration of 0.05 mg mL^−1^ in 10 mM Tris-HCl buffer, pH 7.8, using a 1.0 cm path length cell. Change in the fluorescence spectra of the proteins in the presence of increasing amount of Ca^2+^ was evaluated by measuring the emission upon incubation of the protein with CaCl_2_ in increasing protein to salt molar ratio for 24 hours at 4 °C.

### Statistical analysis

Graphics were created with GraphPad Prism 4.0 for Windows (Graphpad Software, San Diego, CA, USA). Statistical analyses were performed with Past software ver. 3.11 (http://folk.uio.no/ohammer/past/terms.html). The test used and the number of experiments were reported in the figure legends.

## Electronic supplementary material

Supplementary Figures 1, 2, 3.

Dataset 1, Dataset 2.
