# Supplementary material for: Biotic and environmental stress induces nitration and changes in structure and function of the sea urchin major yolk protein toposome
Source: Sci Rep. 2018 Mar 15;8:4610. doi: 10.1038/s41598-018-22861-1 (PMC5854732; doi:10.1038/s41598-018-22861-1)
Supplement: Supplementary file 1 — Supplementary Figures 1, 2, 3. [file 41598_2018_22861_MOESM1_ESM.pdf]

Biotic and environmental stress induces nitration and changes in structure and function of the sea urchin major yolk protein toposome

Immacolata Castellano, Oriana Migliaccio, Giarita Ferraro, Elisa Maffioli, Daniela Marasco, Antonello Merlino, Adriana Zingone, Gabriella Tedeschi & Anna Palumbo

Supplementary Figure 1. Multiple sequence alignment of *P. lividus* toposome with homologous counterparts. *P. lividus* (_PARLI) was aligned with the following sequences: *Pseudocentrotus depressus* (_PSEDP), *Hemicentrotus pulcherrimus* (_HEMPU), *Mesocentrotus nudus* (_MESNU), *Strongylocentrotus purpuratus* (_STRPU), *Tripneustes gratilla* (_TRIGR), *Lytechinus variegatus* (_LYTVA) *and Stichopus japonicus* (_STIJA). All sequences show a typical and conserved transferrin domain whose residues are indicated in bold. In red the residues of toposome from *Paracentrotus lividus* found nitrated in C (control pre-bloom animals), T0 (sample collected at bloom), T1 (after stabulation for 56 days) and T2 (after stabulation for 92 days); in blue the residues nitrated only in sea urchin developing embryos after treatment of fertilized eggs with cadmium and manganese.

```
TR|Q6WQT5|Q6WQT5_PARLI         MRVAILLCLVASAVAAPSS--MWGVREGSCPPPPDAETQMSATRCSYVYGL-IWDYTCDQ 57
TR|Q964G1|Q964G1_PSEDP         MRAAILFCLVASSMAVPSG--SLGWRSGTCPPQPDDDVLREATRCSYVYGL-VWDYTCDS 57
TR|Q7Z1Y6|Q7Z1Y6_HEMPU         MRAAILFCLVASSMAIPSG--LSGWRSGTCPPQPSEQDMLEATRCSYVYGL-IWDYTCDS 57
TR|Q3YL94|Q3YL94_MESNU         MRALVLFCLVASSVAVPSG--SLGRRPGTCPPQPNNDDMLTATRCSYVYGL-TWDYTCDN 57
TR|A0A1B4Z330|A0A1B4Z330_MESNU MRALILFCLVASSVAVPSG--SLGRRPGTCPPQPNEDDMLGATRCSYVYGL-TWDYTCDN 57
SP|P19615|MYP_STRPU            MRAAILFCLVASSMAVPSG--SLGSRPGTCPPQPSDQVMIEATRCSYVYGL-TWDWNCNS 57
TR|Q8WQY0|Q8WQY0_TRIGR         MRAAILFCLVASSVAFG----V-WERPGKCPPRPDEATIREATRCSSAYGLLRWDYPCDQ 55
TR|Q8T3T1|Q8T3T1_LYTVA         MRVALLLCLVAAAHAVP----PLYARDGLCPTEAEVQDPERLLRCT------GYDWGCPG 50
TR|C4TQH8|C4TQH8_STIJA         MKYLLLFCLAVGAFAS-----INIEREGECPTDITNVDS-------TLYGVCNDECDVDS 48
TR|C4TQH7|C4TQH7_STIJA         MKYLLLLSVIGLAFAGPTPVTKDVVTAGTCPVDHV---F-------PTRGMCEPDCLDDR 50
                               *:  :*:.:   : *            * **                       :

TR|Q6WQT5|Q6WQT5_PARLI         PGQENHKCCEYD-DVRICVPPVQESGEVGA-SNPVDQVRSEDQIRVAIEKTKDLVRKVGR 115
TR|Q964G1|Q964G1_PSEDP         PGQENYKCCQYENDIRICVPPVPSD-DVEVGMQERSQVQTEDQVRQAILKTQDFIRKVGL 116
TR|Q7Z1Y6|Q7Z1Y6_HEMPU         PGQENYKCCQYENDIRICVPPMPEV---ELGVQEPSQVHSVDQVRQAILKTQDFIHKVGL 114
TR|Q3YL94|Q3YL94_MESNU         PGQENYKCCQYENDIRICVPPIPDN-DVEVNVQGHSQVQTEDQVRQAILKTQDFIRKVGL 116
TR|A0A1B4Z330|A0A1B4Z330_MESNU PGQENYKCCQYENDIRICVPPIPDN-DVEVNVQGHSQVQTEDQVRQAILKTQDFIRKVGL 116
SP|P19615|MYP_STRPU            QGQENYKCCQYENDIRICVPPIPADVDEEVGVEQPS--QSVDQVRQAIQKTQDFIRKVGL 115
TR|Q8WQY0|Q8WQY0_TRIGR         PGQETYKCCEYGDDIRICVPPVLNNEDQV--VQGPKPVETPDQVRQAVLKTQDFIRKVGL 113
TR|Q8T3T1|Q8T3T1_LYTVA         ----VKKCCQVG-ELQVCVDPVPVQHAVQRPMHLVKNLTEEEFITMRLRETRDFLQKLER 105
TR|C4TQH8|C4TQH8_STIJA         SCSDQLHKCCATNCGRRCVNPMRVTPVTEEEENKTR-EEITEMLTTILSERRDLIKKLDM 107
TR|C4TQH7|C4TQH7_STIJA         YCQEGLKCCMTANCGLKCLAPVVQN--APENITKTK-QEITELLTTILEERQDLIEKLEK 107
                                     : *        *: *:                   : :   : : :*::.*:
                                                                    Y154
```

```
                                                                      |
TR|Q6WQT5|Q6WQT5_PARLI         YAPPQEQDRTP-VTPNTIRWCVSNQCQMTKCQRMVNEFTYDVNMVPRKEWKCVQATCQEQ 174
TR|Q964G1|Q964G1_PSEDP         YPAPEQRLRTT-PTPDTIRWCVSSPCQMTKCQRMVNEFTYSPNMVPRKEWKCTQATSQEQ 175
TR|Q7Z1Y6|Q7Z1Y6_HEMPU         YPAPEQRLRTT-PTPDTIRWCVSSPCQMTKCQRMVSEFTYSPNMVPRKQWTCTQATSQEQ 173
TR|Q3YL94|Q3YL94_MESNU         YPAPEQRLRTT-PSPDTIRWCVSSPCQMTKCQRMVNEFTYSHNMVPRKEWKCTQATSQEQ 175
TR|A0A1B4Z330|A0A1B4Z330_MESNU YPAPEQRLRTT-PSPDTIRWCVSSPCQMTKCQRMVNEFTYSPNMVPRKEWKCTQATSQEQ 175
SP|P19615|MYP_STRPU            YPAPDQRRRTT-PTPDTVRWCVSSRCQMTKCQRMVSEFTYSPNMVPRKQWKCTQATSQEQ 174
TR|Q8WQY0|Q8WQY0_TRIGR         YPAPEQELRYP-VNPNVIRFCVSSTCQMTKCRRMVSEFTFNPNMAPRKDWRCIQADSQEQ 172
TR|Q8T3T1|Q8T3T1_LYTVA         YSTPPLEKITV-VEPDTIRWCVSSPCQMKKCQRMVNEFTYKSQLTPKKIWSCVKAESQEQ 164
TR|C4TQH8|C4TQH8_STIJA         YPPPLARFIMMKNRTDVVRMCVTTPCELRKCQRIAQTMTYK--VTPRKEWFCQLATTTEQ 165
TR|C4TQH7|C4TQH7_STIJA         FPPPMLRFIAMKNRTDVMRMCVTSPCELRKCQRLATELTYN--VVPRKEHFCQVATSTKQ 165
                               :  *  .        :.:* **:. *:: **:*:.  :*:.  :.*:*   *  *   :*

TR|Q6WQT5|Q6WQT5_PARLI         CMFWIEQGWADIMTTREGEVYTANTTFNLKPIAYETTINDEQPEVQILKHYQNITFALKS 234
TR|Q964G1|Q964G1_PSEDP         CMFWIEQGWADIMTTREGQVYSANTTFNLKPIAYETTITDQQPEVQKLKHYQNVTFALKS 235
TR|Q7Z1Y6|Q7Z1Y6_HEMPU         CMFWIEQGWADIMTTREGQVYSANTTFNLKPIAYETTIND-QPEIQTLKHYQNVTFALKS 232
TR|Q3YL94|Q3YL94_MESNU         CMFWIEQGWADIMTTREGQVYSANTTFNLKPIAYETTITDQQPEIQLLKHYQNVTFALKS 235
TR|A0A1B4Z330|A0A1B4Z330_MESNU CMFWIEQGWADIMTTREGQVYSANTTFNLKPIAYETTITDEQPEIQLLKHYQNVTFALKS 235
SP|P19615|MYP_STRPU            CMFWIEQGWADIMTTREGQVYSANTTFNLKPIAYETTINDQQPEIQILKHYQNVTFALKS 234
TR|Q8WQY0|Q8WQY0_TRIGR         CMFWIEQGWADIMTAREEQVYVANTTFNLKPIAYETTINNDLPE--TLKHYQNVTFALKS 230
TR|Q8T3T1|Q8T3T1_LYTVA         CMFWIERGWADIMTTRGEEVYVANTTFKLEPIAYEKTIKPQEQEVLPLKHYQNVTFALKM 224
TR|C4TQH8|C4TQH8_STIJA         CLFWAERGWTDTVMARENKIFVAVDKFNVTSLAYEKNKNAP-TE--IEKKVQNITLALTL 222
TR|C4TQH7|C4TQH7_STIJA         CLFWVERGWADLVVARDLDIYHAKTKFNVTAFAQETLKNENLAL--IEKVSQNITLAVTM 223
                               *:** *:**:* : :*  .:: *  .*::  :* *.  .         *  **:*:*:.

TR|Q6WQT5|Q6WQT5_PARLI         SRLVNPNTFAELQDKTTCHAGINM-----PASFADPVCHLIKEGVIPVTGNYVESFSDFV 289
TR|Q964G1|Q964G1_PSEDP         SRLVNPNTFAELRDKTTCHAGIDM-----PASFADPVCNLIKEGVIPVTGNHIESFSDFV 290
TR|Q7Z1Y6|Q7Z1Y6_HEMPU         SRLVNPNTFAELRDKTTCHAGIDM-----PASFADPVCNLIKEGVIPVTGNYIESFSDFV 287
TR|Q3YL94|Q3YL94_MESNU         SRLVNPNTFAELRDKTTCHAGIDM-----PASFADPVCNLIKEGVIPVTGNHVESFSDFV 290
TR|A0A1B4Z330|A0A1B4Z330_MESNU SRLVNPNTFAELHDKTTCHAGIDM-----PASFADPVCNLIKEGIIPVTGNHVESFSDFV 290
SP|P19615|MYP_STRPU            SRLVNPNTFSELRDKTTCHAGIDMPDFDMPASFADPVCNLIKEGVIPVTGNYIESFSDFV 294
TR|Q8WQY0|Q8WQY0_TRIGR         SRLINPNTFSELRDKTTCHAGIDM-----PASFADPVCNLIKEGVIPVTGNYIESFADFV 285
TR|Q8T3T1|Q8T3T1_LYTVA         SHQIAPMTWSEIRDKTTCHAGADF-----PASFKAPVCRLIAEEVINKTGDYAESVADFV 279
TR|C4TQH8|C4TQH8_STIJA         T-TSNIETFHHLMGRKVCSAGVNI-----TSAFITPMCNLIYKDVMLATGDVVESSADFF 276
TR|C4TQH7|C4TQH7_STIJA         R-QSHIHTLNQLEGAPICSAGINN-----TASFISPVATLISKGIIPITGSVLESAADFF 277
                                      *  .: .   * ** :       ::*  *:. ** : ::  **.  ** :**.
                                                Y308        Y321 Y323
                                                  |            |  |
TR|Q6WQT5|Q6WQT5_PARLI         QESCIPGVLNKT-YNKNGTYPLSLVSLCEDQQYEYSGIKGALKCLDSGKGQVTFVDQKVI 348
TR|Q964G1|Q964G1_PSEDP         QESCVPGVLNKT-YNKNGTYPLSLVTLCEDQQYEYSGIKGALRCLDSGKGQVTFVDQKVI 349
TR|Q7Z1Y6|Q7Z1Y6_HEMPU         QESCVPGVLNKT-YNKNGTYPLSLVTLCEDQQYEYSGIKGALRCLDSGKGQVTFVDQKVI 346
TR|Q3YL94|Q3YL94_MESNU         QESCVPGVLNKT-YNKNGTYPLSLVTLCEDQQYEYSGIKGALRCLESGKGQVTFVDQKVI 349
TR|A0A1B4Z330|A0A1B4Z330_MESNU QESCVPGVLNKT-YNKNGTYPLSLVTLCEDQQYEYSGIKGALRCLDSGKGQVTFVDQKVI 349
SP|P19615|MYP_STRPU            QESCVPGVLNMT-YNKNGTYPLSLVTLCEDQQYKYSGIKGALSCLESGKGQVTFVDQKVI 353
TR|Q8WQY0|Q8WQY0_TRIGR         QESCLPGVLNKT-YNKNGTYPRTLISLCEDRQAEYSGIKGALKCLDSGKGQVTFVDQKEI 344
TR|Q8T3T1|Q8T3T1_LYTVA         EESCVPGILNKT-YNKNMTYPMNLVSLCEKDQLKYSGVEGSLKCLRSGKGQVTFVDHKIV 338
TR|C4TQH8|C4TQH8_STIJA         GKMCVPGILNKT-FDKNETYPEKLTTACHNLETEYTGIKGSLKCIKSGHGEVVFVDSKVV 335
TR|C4TQH7|C4TQH7_STIJA         GDMCVPGALNKTTVNTNETLPEKFLTGCNQRVDEFTGIVGSLRCVSSVNG-VAFVDHKIV 336
                                . *:** ** *  :.* * * .: : *..   :::*: *:* *: * :* *.*** * :

TR|Q6WQT5|Q6WQT5_PARLI         KKIMSDEVERENYMVVCQDESRPLDEEIFTDVTCHVGHTARPTIFINKNNSE--EADVKN 406
TR|Q964G1|Q964G1_PSEDP         KKIMSDPNERNNYQVVCRDESRLLDEEVFTDGTCHIGHTARPTIFINKNNTQQKERDMKD 409
TR|Q7Z1Y6|Q7Z1Y6_HEMPU         KKIMSDPSVRDNFQVVCRDESRPLDEEIFADVTCHVGHTARPTIFINKNNTQQKEIDIKS 406
TR|Q3YL94|Q3YL94_MESNU         KEIMSDIDLRNNYQVVCRDESRLLDQEIFRDSTCHVGHTARPTIFINKNNTQQKELDMKT 409
TR|A0A1B4Z330|A0A1B4Z330_MESNU KEIMSDINLRNNYQVVCRDESRLLDQEIFRDSTCHVGHTARPTIFINKNNTHQKEMDLKA 409
SP|P19615|MYP_STRPU            KKIMSDPNVRDNFQVVCRDESRPLDEEIFTDVTCHVGHTARPTIFINKNNTQQKETDIKT 413
TR|Q8WQY0|Q8WQY0_TRIGR         MRIMNDENVRDNYMVVCRDESRPLEREIFDDVTCHVGHTARPTIFINRNNTQQQERDFKT 404
TR|Q8T3T1|Q8T3T1_LYTVA         KKLMMDENERTTTWLSARARASHLTRPSLRDTTCHVGHVANPTVFIARNNTEVKKVEIKD 398
TR|C4TQH8|C4TQH8_STIJA         KELD--ERFAGVFKLVCEDQSLPLSQ--WEQTKCHLGYTPRPVLFLNPERNVTYKNELKE 391
TR|C4TQH7|C4TQH7_STIJA         KEMMENTEYNDNFRLVCEGENKPLSS--WVEKPCQIGYTPRPILFVNPERNTTYIQELTT 394
                                .:           : .. .   *      :  *::*:. .* :*:  :..     :..
                                    Y418
                                       |
TR|Q6WQT5|Q6WQT5_PARLI         LIKKMMQIYGNTDPTRAFNIFDSSVYDCDTCKKSGRPLNQNVIFLDESNTLKIIDDSKA- 465
TR|Q964G1|Q964G1_PSEDP         LVEKMMELYGNTDPTVQLNIFDSSVYDCGKCQRTGKPLNKNLIFLEESNTIKKLDDSKV- 468
TR|Q7Z1Y6|Q7Z1Y6_HEMPU         LVVKMMELYGNTDRNVQFNIFDSSVYDCGKCQKTGKPRNKNLIFLEESNTIKIVDDSKV- 465
TR|Q3YL94|Q3YL94_MESNU         LVEKMMELYGNTDPTVQFNIFDSSAYDCGKCQKTGKPLNKNLIFLEESNTMKIVDDSKV- 468
TR|A0A1B4Z330|A0A1B4Z330_MESNU LVEKMMELYGNTDPTVQFNIFDSSAYDCGKCQKTGKPLNKNLMFLEESNTMKIVDDSKV- 468
SP|P19615|MYP_STRPU            LVVKMMELYGNTDRDVNFNIFDSSVYDCGKCQMTGKPLNKNLIFLEESNTMKIVDDSKV- 472
TR|Q8WQY0|Q8WQY0_TRIGR         LVQKMAEIYRMTDVYDRFNLFDSSVYTCDKCRKDGRLQNKNLIFLDESNTLEILDDAKV- 463
TR|Q8T3T1|Q8T3T1_LYTVA         LLRKILSLYSTTNPEIELNIFDSSVYECEPCSWTGKRTNKDLIFLEESTTLKIIDPSKV- 457
TR|C4TQH8|C4TQH8_STIJA         IILEAGK-----MKTPTVDLFNSSDYVCI------KEAPKDLIFMDENTNLEFLEDPFSL 440
TR|C4TQH7|C4TQH7_STIJA         IMTNVVK--KNTGKVGSLNIFNSTEIVCG------NEPNKNLIFMDENNAFTAINESIAE 446
                               :: :  .          .::*:*:   *       .   ::::*::*.. :  ::
```

```
                                  Y466 Y471
                                     |    |
TR|Q6WQT5|Q6WQT5_PARLI           ---YAHHVYAAYNTCSKLTPKPRAKICVTNVTEYDACRRFKGIAENVPEVKNVAWGCILA 522
TR|Q964G1|Q964G1_PSEDP           ---YAGEVYAAYNICSKLVPKPRAKICVTNVTEYEACRRFKGIAENIPEVKKVAWGCVLA 525
TR|Q7Z1Y6|Q7Z1Y6_HEMPU           ---YAGEVYAAYNVCSKLVPKPRAKICVTNVTEYEACRRFKGIAENIPEVKNVAWGCVLA 522
TR|Q3YL94|Q3YL94_MESNU           ---YAGEVYAAYNICSKLVPKPRAKICVTNVTEYEACRRFKGIAENIPEVKKVAWGCVLA 525
TR|A0A1B4Z330|A0A1B4Z330_MESNU   ---YAGEVYAAYNICSKLVPKPRAKICVTNVTKYEACRRFKGIAENIPEVKKVAWGCVLA 525
SP|P19615|MYP_STRPU              ---YAGEVYAAYNVCSKLVPKPRAKICVTNVTEYEACRRFKGIAENIPEVKKVAWGCVLA 529
TR|Q8WQY0|Q8WQY0_TRIGR           ---FAGEVYAAYNTCSQLVPKPRAKICVTNVTEYEACRRFKGIAENIPQVKNVAWGCVLA 520
TR|Q8T3T1|Q8T3T1_LYTVA           ---YAEKFFQSFNTCVQLQPKPRAKICVTTVEKYNSCLAFKDIAEKFMKVKDVAWGCVLA 514
TR|C4TQH8|C4TQH8_STIJA           P--AVQEYMKVFNTCQALTPKPRAKICTTTPVQYQKCITMKKVFQTDIELRNISWGCVLA 498
TR|C4TQH7|C4TQH7_STIJA           QRIETKSYFRTYNICESLVPKPTVRFCVTKPVEYNKCLTMKHVFEKHEKLSHIAWGCVHA 506
                                     .      :* *   * *** .::*.*.   :*: *   :* : :.   :: .::***: *

                                                                                      Y574
                                                                                        |
TR|Q6WQT5|Q6WQT5_PARLI           NSSMECMQAVHNNTADLYKACPVETFIAGKEFLLDPLMSVHRNNSVTLNHTY-----TRT 577
TR|Q964G1|Q964G1_PSEDP           NSSLECMQAVHNNTADLFKANPMETFIAGKEFLLDPLMSVHRNDSVSRNHTQ-----TRT 580
TR|Q7Z1Y6|Q7Z1Y6_HEMPU           NSSIECMQAVHNNTADLFKANPMETFIAGKEFLLDPLMSVHRNDSVTRNHTY-----TRT 577
TR|Q3YL94|Q3YL94_MESNU           NSSLECMQAVHNNTADLFKANPMETFIAGKEFLLDPLMSVHRNDSVTKNHTY-----TRT 580
TR|A0A1B4Z330|A0A1B4Z330_MESNU   NSSLECMQAVHNNTADLFKANPMETFIAGKEFLLDPLMSVHRNDSVTKNHTY-----TRT 580
SP|P19615|MYP_STRPU              NSSIECMQAVHNNTADLFKANPMETFIAGKEFLLDPLMSVHRNDSVTMNHTY-----TRT 584
TR|Q8WQY0|Q8WQY0_TRIGR           NSSMECMKAVHDNTADLFKANPQETFIAGKEFLLDPLMSVHRNDSVTLNHTY-----TRT 575
TR|Q8T3T1|Q8T3T1_LYTVA           NSSLECMQAVHNNTADLFQADPVETFIAGKEMLLEPLMSVHKNFSISPNHTF-----TRT 569
TR|C4TQH8|C4TQH8_STIJA           RTEMDCMRNVLNGTADLYSGDVKEIFTAGNDFQLQPMMIQDENLRFQKSLFHNATVKSYT 558
TR|C4TQH7|C4TQH7_STIJA           PTSMECMRNVLNGTAEIFSGNVEEIFIGGRDFLLQPIMMQDPVMRESINDYVNKVFGTHS 566
                                  :.::**: * :.**:::..    * * .*.:: *:*:*   .       .       : :

                                           Y589                 W607                Y628
                                             |                    |                    |
TR|Q6WQT5|Q6WQT5_PARLI           LAVIKRSSLSKYPDLLNVPEGQPKYIKDLWKLKICSAGLRNFSAFSNPIGYLLANGTIPR 637
TR|Q964G1|Q964G1_PSEDP           LAVIKRSSLAKFPDLLNVPEGQPKYIKDLWKLKICSAGLKNFSAFHSAIGYLLANGTIPR 640
TR|Q7Z1Y6|Q7Z1Y6_HEMPU           LAVIKRSSLAKFPGLLNVPEGQPKYIKDLWKLKICSAGLKNFSAFHSPIGYLLANGTIPR 637
TR|Q3YL94|Q3YL94_MESNU           LAVIKRSSLAQFPDLLNVPEGQPKYIKDLWKLKVCSAGLKNFSAFHSAIGYLLANGTIPR 640
TR|A0A1B4Z330|A0A1B4Z330_MESNU   LAVIKRSSLAQFPDLLNVPEGQPKYIKDLWKLKVCSAGLKNFSAFHSAIGYLLANGTIPR 640
SP|P19615|MYP_STRPU              LAVIKRSSLAKFPGLLSVPEGQPKYIKDLWKLKICSAGLKNFSAFHSPIGYLLANGTIPR 644
TR|Q8WQY0|Q8WQY0_TRIGR           LAVIKRSSLNQFPDILNVPEGQPKYIKDLYKLRICSAGLKNFSAFANPIGYLLANGTIPR 635
TR|Q8T3T1|Q8T3T1_LYTVA           LAVIKASSLSQFEEIMRTPEDQPKYIKDLWKLKTCHAGLKNVSSFHNPIGWLLSNGTIPR 629
TR|C4TQH8|C4TQH8_STIJA           IAVMKKSKFWK-------KFGEDTKVVNIRNLTVCSPDIKSVPNFHLPIGHLLSNGVIPR 611
TR|C4TQH7|C4TQH7_STIJA           VSVMKKSKLWN-------KFGQDTKFLNIRNLTVCSAGINKVESFHLPVGRMLSTNVMPR 619
                                 ::*:* *.: :           .: . . :: :*  *  .:..   *   :* :*:...:**

TR|Q6WQT5|Q6WQT5_PARLI           IGSVFESVNRYFQATCIPEIEPETFRWDSDLLLGREMNWGFPTLNMYNFTGQEWMLWNTP 697
TR|Q964G1|Q964G1_PSEDP           IGSVFESVNRYFQATCIPEIEPETFRLDSDLLLGREMNWGFSSLNMFNFTGQEWLLWNTP 700
TR|Q7Z1Y6|Q7Z1Y6_HEMPU           IGSVFESVNRYFQASCVPEIEPETYRLDSDLLLGREMNWGFSSLNMFNFTGQEWLLWNTP 697
TR|Q3YL94|Q3YL94_MESNU           IGSVFESVNRYFQATCIPEIEPETWRLDSDLLLGREMNWGFSSLNMFNFTGQEWLLWNTP 700
TR|A0A1B4Z330|A0A1B4Z330_MESNU   IGSVFESVNRYFQATCIPEIEPETWRLDSDLLLGREMNWGFSSLNMFNFTGQEWLLWNTP 700
SP|P19615|MYP_STRPU              IGSVFESVNRYFQATCVPEIEPETWRLDSDLLLGREMNWGFSSLNMYNFTGQEWLLWNTP 704
TR|Q8WQY0|Q8WQY0_TRIGR           IGSVFESVNRYFQSTCIPEIEPERFRFDSDLLLGRELNWGFSTLNMYNFTGQEWLLWNTP 695
TR|Q8T3T1|Q8T3T1_LYTVA           IGSVFESINRYFKSSCLPDVSDQD-IWMKDLLLGHEHNWGYPTLNMYNFTGQDWLLWNTP 688
TR|C4TQH8|C4TQH8_STIJA           IGSVFESVSRFYKSVCLPGAAPVDWTWDSDLILGHEVNWGIPGLSFYNFTGFDWFIWNAP 671
TR|C4TQH7|C4TQH7_STIJA           IGSVFESVNRFFKSACIPGATPVSYKHDLDLVIGHELNWGITGLSFYNFTGFDWFIWNAP 679
                                 *******:.*:::: *:*             **::*:* ***    *.::**** :*::**:*

TR|Q6WQT5|Q6WQT5_PARLI           ATWNFLTYNRKVSKGVNVKQLLELKRLNLTSHLNRNDPNTP---------MNVELLDDLV 748
TR|Q964G1|Q964G1_PSEDP           ATWNFLTYNRKVSTGLDIKKLIELKKQNLTSHVFNRNLSTS---------LNVELLDDLI 751
TR|Q7Z1Y6|Q7Z1Y6_HEMPU           ATWNFLTYNRKVSSGLDIKKLIELKKQNLTSHIFNHNLSSP---------MNVELLDDLV 748
TR|Q3YL94|Q3YL94_MESNU           ATWNFLTYNRKVSNGLDIKKLLELKKQNLTSHIFNHNLSTP---------MNVELLDDLV 751
TR|A0A1B4Z330|A0A1B4Z330_MESNU   ATWNFLTYNRKVSNGLDIKKLLELKKQNLTSHIFNHNLSTP---------MNVELLDDLV 751
SP|P19615|MYP_STRPU              ATWNFLTYNRKVSTGLDIKKLIELKKQNLTSHIFNQNLSSP---------RNVELLDDLV 755
TR|Q8WQY0|Q8WQY0_TRIGR           ATWNFLTYNRKVSNGLDIKKLIELKRQNLTSHIFNQNLT-S---------ANVELLDDLV 745
TR|Q8T3T1|Q8T3T1_LYTVA           ATWNYLTYNRKAARSLNLAKLVELQKLNLTNPLLETVPEDI---------SSLHI----- 734
TR|C4TQH8|C4TQH8_STIJA           HTWTFYNMNRKTPGTLK-----EFMKNRMLIPLLEGKLTVPEGFN--PDTFDYSILDDVL 724
TR|C4TQH7|C4TQH7_STIJA           HTWTFYNYNRKTPGTLQ-----RFMKNRLLVPLLEGKLSLLMSLTLTPSTTASLMM---S 731
                                   **.: . ***.    :.      .: : .:   : .                   :

TR|Q6WQT5|Q6WQT5_PARLI           GVEGLSDLVKGLQQTMGPE-------GRDKMA-------------------------QLR 776
TR|Q964G1|Q964G1_PSEDP           GVEGISDLVKGLQDTMGLE-------GRQKMS-------------------------MLR 779
TR|Q7Z1Y6|Q7Z1Y6_HEMPU           GVEGISDLVKGVQDTITPE-------GREKMN-------------------------MLR 776
TR|Q3YL94|Q3YL94_MESNU           GVEGISDLVKGLQDTTGPE-------GRKKMN-------------------------MLR 779
TR|A0A1B4Z330|A0A1B4Z330_MESNU   GVEGISDLVKGLQDTTGPE-------GRKKMN-------------------------MLR 779
SP|P19615|MYP_STRPU              GVEGISDLVKGVQDTIGPE-------GKQKMN-------------------------MLR 783
TR|Q8WQY0|Q8WQY0_TRIGR           GVDGLSDLIKGLQDSISPE-------GKQRLN-------------------------LIR 773
TR|Q8T3T1|Q8T3T1_LYTVA           -----SSSVKVALNSIIPC-------SSNF---------------------------SP 754
TR|C4TQH8|C4TQH8_STIJA           SVEGLGDILNDIPDEIRDSLVDIRTKRVDRKEKFRELYEEKTGDAFVSLRDRRLGKLTLR 784
TR|C4TQH7|C4TQH7_STIJA           SALKTRDILSDIPDKIRDRLVDMRVSNKDKMSTFKDLYEDRTGTEFVRPKDKRLSKQTLR 791
```

```
                                         . :.   :              .

TR|Q6WQT5|Q6WQT5_PARLI          DRLGNAFPNFEALTPLSDKIEM--------INKMREAHEN----------RIQSRDTPFG 818
TR|Q964G1|Q964G1_PSEDP          DRLSNSYPNFDAVRTLSDKVDI--------INKMKEARQI----------RLKNQDHPFG 821
TR|Q7Z1Y6|Q7Z1Y6_HEMPU          DRLSNSFPNFEAVRTLSDKVDI--------INKMKDARQQ----------RLQNQDHPFG 818
TR|Q3YL94|Q3YL94_MESNU          DRLSNSFPNFDAVRTLSDKVDI--------INKMKDARQS----------RLQNQDHPFG 821
TR|A0A1B4Z330|A0A1B4Z330_MESNU DRLSNSFPNFDAVRTLSDKVDI--------INKMKDARQS----------RLQNQDHPFG 821
SP|P19615|MYP_STRPU             DRLSNSFPNFEAVRTLSDKVDI--------VNKMKDARQQ----------RLQNKDHPFG 825
TR|Q8WQY0|Q8WQY0_TRIGR          DRLSNSFPNFEGVRTLSDKVDM--------INRMQENRRN----------RIQNQDTPFA 815
TR|Q8T3T1|Q8T3T1_LYTVA          YILSPPVP---------------------------------------------------- 762
TR|C4TQH8|C4TQH8_STIJA          GFLQQSFQNMEDEWGIFDDVNAPVLSSKSVGKGMTGGKVSNLRDILRGAATTADDNTVIG 844
TR|C4TQH7|C4TQH7_STIJA          SYLQQSFTNMQDEWGIFENVNNPVMTPNKPVKSMTGGKVTNVRRFFDEHVKETDPNTIIG 851
                                  *

                                                                             Y864
                                                                              |
TR|Q6WQT5|Q6WQT5_PARLI          NLVQ---ELFQGQLMVDIFGKLLELRSDKISTLEEIISHVKTIPFLTDYKDEEITTVLKH 875
TR|Q964G1|Q964G1_PSEDP          NVIQ---ETFQGHLMVDVFSKLLELRSDKINTLEEIISHVKTIPYLTDFKDVEITTVIKH 878
TR|Q7Z1Y6|Q7Z1Y6_HEMPU          NVIQ---ETFQGHLMVDVFSKLLELRSDKISTLEEIISHVKTIPYLTDFKDVEITTVLKH 875
TR|Q3YL94|Q3YL94_MESNU          NVIQ---ETFQGHLMVDVFSKLLELRSDKISTLEEIISHVKTIPYLTDFKDVEITTVIKH 878
TR|A0A1B4Z330|A0A1B4Z330_MESNU NVIQ---ETFQGHLMVDVFSKLLELRSDKISTLEEIISHVKTIPYLTDFKDVEITTVIKH 878
SP|P19615|MYP_STRPU             NVIQ---ETFQGHLMVDVFSKLLELRSDKISTLEEIISHVKTIPYLTDFKDVEITTVLKH 882
TR|Q8WQY0|Q8WQY0_TRIGR          DYIQ---GKFGGELMVDIFSKLLELRSDKIATLEEIISHVKSIPYLTDFKDEEITTVIKH 872
TR|Q8T3T1|Q8T3T1_LYTVA          ------------------------------------------------------------
TR|C4TQH8|C4TQH8_STIJA          NLLKDYDSEYTVPVISRIFSKLLNERFETLDGLAKTLEILHRVPTMSSIRGTDY-EWLKH 903
TR|C4TQH7|C4TQH7_STIJA          NLLMDYDSEYTVPLITKIFAKVLNQRFDKLFGLADSLSILHGVPTMSSVHDEQY-EWIKH 910

                                      Y885                         W915
                                       |                            |
TR|Q6WQT5|Q6WQT5_PARLI          PAIMSYVEIYFPRLSQTFVEPFDNVELREREFNRYTNPLWLSPKINNFIEMVKKHQAEIT 935
TR|Q964G1|Q964G1_PSEDP          PAIMSYVEIYFPRLSQTFVEPFDNVELREREFNPYTNPLWLSPKIHTYLEMVKKHQTEIT 938
TR|Q7Z1Y6|Q7Z1Y6_HEMPU          PAILSYVEIYFPRLSQTFVEPFDNVELREREFNRYTNPLWLSPKIQTYLDLVQKHQTEIT 935
TR|Q3YL94|Q3YL94_MESNU          PAIMSYIEIYFPRLSQTFVEPFDNVELREREFNRYTNPLWLSPKVHTYLEMVKKHQTEIT 938
TR|A0A1B4Z330|A0A1B4Z330_MESNU PAIMSYIEIYFPRLSQTSVEPFDNVELREREFNRYTNPLWLSPKVHTYLEMVKKHQTEIT 938
SP|P19615|MYP_STRPU             PAIMSYVEIYFPRLSQTFVEPFDNVELREREFNRYTNPLWLSPKVHTYLDLVKNHQTEIT 942
TR|Q8WQY0|Q8WQY0_TRIGR          PAIMSYVEIYFPRLAQTFVEPFDNAELREREFNRYTNPLWLSPRIDTYLDVIKKHQNEIT 932
TR|Q8T3T1|Q8T3T1_LYTVA          ------------------------------------------------------------
TR|C4TQH8|C4TQH8_STIJA          PAVQSYLKIYAPRLISFHSDLYTSEELAKTQFSRYLNPIWLSPTFKDFLDVTKTHMTKLT 963
TR|C4TQH7|C4TQH7_STIJA          PAVQAFVRIYAPRLATYYSDLYTNSELNSMVHSRYHNPLWLTPTYPEFLDTMKTHMTDLI 970


TR|Q6WQT5|Q6WQT5_PARLI          KTCNS--------NLPLNFKGYEGSLRCLKSGVAD-MAFFDEQ-----TLRDQDLLSRVG 981
TR|Q964G1|Q964G1_PSEDP          KTCNS--------NLPLNFKGYEGSLRCLKSGVAD-MAFFDEQ-----TLRDQDLLSRVG 984
TR|Q7Z1Y6|Q7Z1Y6_HEMPU          KTCNS--------NLPLNFKGYEGSLRCLKSGVAD-LAFFDEQ-----TLRDTDLLSRVG 981
TR|Q3YL94|Q3YL94_MESNU          KTCNS--------NLPLNFKGYEGSLRCLKSGVAD-MAFFDEQ-----TLRDEDLLSRVG 984
TR|A0A1B4Z330|A0A1B4Z330_MESNU KTCNS--------NLPLNFKGYEGSLRCLKSGVAD-MAFFDEQ-----TLRDEDLLSRVG 984
SP|P19615|MYP_STRPU             KTCNS--------NLPLNFKGYEGALRCLKSGVAD-LASSTSRPSVTRTLRDTDLSTGW- 992
TR|Q8WQY0|Q8WQY0_TRIGR          QTCNS--------NLPLKFNGYEGSLRCLKSGAAD-LAFFDEQ-----TLRDQDLLSRVG 978
TR|Q8T3T1|Q8T3T1_LYTVA          ------------------------------------------------------------
TR|C4TQH8|C4TQH8_STIJA          EMCRGYGDRQGVGNSHEPFYGNEGALRCIEDTEEGDIAFIDTK-NFA------------T 1010
TR|C4TQH7|C4TQH7_STIJA          HICQGFGEHKGELSDEEPFYGLAGSIECIADGSEGDIAFVELE-SLVKEL------PKMG 1023

                                                                          Y1023   W1031
                                                                            |       |
TR|Q6WQT5|Q6WQT5_PARLI          FTYNDLRLLCPNGQVVEID----VNLDIAKVCNFGEVMNPVLVTSYNTSGSWRWNITKAL 1037
TR|Q964G1|Q964G1_PSEDP          FTYNDLRLLCPNGQVVEID----VNLDITKVCNFGEVMNPVLVTAYNTSGSWRWNITKAL 1040
TR|Q7Z1Y6|Q7Z1Y6_HEMPU          FTYNDLRLLCPNGQVVEID----VNMDIAKVCNFGEVMNPVLVTAYNTSGSWRWNITKAL 1037
TR|Q3YL94|Q3YL94_MESNU          FTYNDLRLLCPNGQVVEID----VNLDIAKVCNFGEVMNPVLVTAYNTSGSWRWNITKAL 1040
TR|A0A1B4Z330|A0A1B4Z330_MESNU FTYNDLRLLCPNGQVVEID----VNLDIAKVCNFGEVMNPVLVTAYNTSGSWRWNITKAL 1040
SP|P19615|MYP_STRPU             VHLQRPPPSLPQRQVVEID----VNMDIAKVCNFGEVMNPVLVTAYNTSGSWRWNITKAL 1048
TR|Q8WQY0|Q8WQY0_TRIGR          FTYNDLRLLCPNGQVVEID----ASLDVAKVCNFGEVMNPVLLTSYNTSGSLRWNITKAL 1034
TR|Q8T3T1|Q8T3T1_LYTVA          ------------------------------------------------------------
TR|C4TQH8|C4TQH8_STIJA          LSTTDYVMVTPLGIVQPINPESIM------NGTFGTVPFPALMTAFNKTGSWRWNVTKAL 1064
TR|C4TQH7|C4TQH7_STIJA          QSILDYVIVTPLGLVKEVTPEMITNITFLRNVTFGVKAFPALLTSFNKTGSWRWNVTKAL 1083


TR|Q6WQT5|Q6WQT5_PARLI          MIAHHSPA-------LPALFGEHTVMGKNFDMLIPIAPLNQSYQTYLGPKPLRSMEAIVK 1090
TR|Q964G1|Q964G1_PSEDP          MIAHQSVA-------LPALFGEGTVLGKDYDMLLPIAPLNQSYQPFLGPKPLRSMEAIVK 1093
TR|Q7Z1Y6|Q7Z1Y6_HEMPU          MIAHQSVA-------LPALFGEGTVMGKDYDMLLPIAPLNQSYQPFLGPKPLRSMEAIVK 1090
TR|Q3YL94|Q3YL94_MESNU          MIAHQSVA-------LPALFGEGTVMGKDYDMLLPIAPLNQSYQPFLGPKPLRSMEAVVK 1093
TR|A0A1B4Z330|A0A1B4Z330_MESNU MIAHQSVA-------LPALFGEGTVMGKDYDMLLPIAPLNQSYQPFLGPKPLRSMEAVVK 1093
SP|P19615|MYP_STRPU             MIAHQSVA-------LPALFGEGTVMGKDYDMLLPIAPLNQSYQPFLGSKPLRSMEAIVK 1101
TR|Q8WQY0|Q8WQY0_TRIGR          MIAHQSVA-------LPALFGEGTVFGKDFDRLLPIAPLNQSYQAFLGPKPLRSMEAVIK 1087
TR|Q8T3T1|Q8T3T1_LYTVA          ------------------------------------------------------------
TR|C4TQH8|C4TQH8_STIJA          LIAQKKYPT--NTSTDYTMYGVDSVFMPETKKMYPVPLNMQTHPTYLGSRLTRAFEALIK 1122
TR|C4TQH7|C4TQH7_STIJA          LDAQKIFSDAQNNEDQYHIFGSASIFAPETVKLAPIPIQDQTYATYLGPNLTRSFEAIIK 1143
```

```
TR|Q6WQT5|Q6WQT5_PARLI         SSSYDWFKDQTGICYGETYTNIVKQRNGTCQAVVKDVTCVGTPRMKKISVGRFGAKQYKM 1150
TR|Q964G1|Q964G1_PSEDP         ASSYDWFKDQTGICYGETYTNIVKQRNGTCQAIVKDVTCVGTPRVKKISVGRLGAKQYKI 1153
TR|Q7Z1Y6|Q7Z1Y6_HEMPU         ASSYDWFKDQTGICYGETYTNIVKQRNGTCQAIVKDVTCVGTPRVKKISVGRFGAKQFKM 1150
TR|Q3YL94|Q3YL94_MESNU         ASSYDWFKDQTGICYGETYTNIVKQRNETCQAIVKDVTCVGTPRMKKISVGRFGAKQYKM 1153
TR|A0A1B4Z330|A0A1B4Z330_MESNU ASSYDWFKDQTGICYGETYTNIIKQRNETCQAIVKDVTCVGTPRMKKISVGRFGAKQYKM 1153
SP|P19615|MYP_STRPU            ASSYDWFKDQTGICYGETYTNIVKQRNETCQAIVKDVTCVGTPRMKKISVGRFGAKQFKM 1161
TR|Q8WQY0|Q8WQY0_TRIGR         ASSYDWFKDQPGICYGETYTNIVKQRNETCQAVVKDVTCVGTPRMKKISVGRFGAKQYKM 1147
TR|Q8T3T1|Q8T3T1_LYTVA         ------------------------------------------------------------
TR|C4TQH8|C4TQH8_STIJA         PSTHDWWKERRHICSGESYTNVIEQRNGTCKAIVKDVTCGGMPRPKVISVGTTENKKPVV 1182
TR|C4TQH7|C4TQH7_STIJA         PSTFDWWKERRHICTGESYTNVIEQRNGTCKAIVKDVTCGGMPRPKVISVGTTENKKPVV 1203

TR|Q6WQT5|Q6WQT5_PARLI         IKMCSRPSKFVRKMADFQCDNGYGYLKPVVTAVACECMPCEERIEYNTSFTQDYMWSKES 1210
TR|Q964G1|Q964G1_PSEDP         IKMCSRPSKFVRKMADFQCDNGFGYLKPVITAVACECMPCEEMIEYNTSFTEDHMWSDVS 1213
TR|Q7Z1Y6|Q7Z1Y6_HEMPU         IKMCSRPSKFVRKMADFQCDNGFGYLKPVITAVACECMPCEEMIEYNTSFTQDHMWSDVS 1210
TR|Q3YL94|Q3YL94_MESNU         IKMCSRPSKFVRKMADFQCDNGFGYLKPVITAVACECMPCVEMIEYNTSFTEDHMWSDRS 1213
TR|A0A1B4Z330|A0A1B4Z330_MESNU IKMCSRPSKFVRKMADFQCDNGFGYLKPVITAVACECMPCVEMIEYNTSFTEDLMWSDRS 1213
SP|P19615|MYP_STRPU            IKMCSRPSKFVRKMADFQCDNGFGYLKPVITAVACECMLCEEMIEYNTSFTEDNMWSDVS 1221
TR|Q8WQY0|Q8WQY0_TRIGR         VKMCSRPSKFVRKMAEFQCDNGYGYLKPVITAIACECMPCEELIEYNASFTQDQMWKNVS 1207
TR|Q8T3T1|Q8T3T1_LYTVA         ------------------------------------------------------------
TR|C4TQH8|C4TQH8_STIJA         VRMCSRPTSFVREMAEFRCDNGYGYLKPVMVPTTCSCVPCDE-IEYKPTWTTDIMWNTTE 1241
TR|C4TQH7|C4TQH7_STIJA         VRMCSRPTSFVREMAEFVCDNGHGYVTPVMVPTTCSCVPCED-IEYIPDWTNDTVWNSTT 1262

                                         Y1222 W1228 Y1235
                                             |     |      |
TR|Q6WQT5|Q6WQT5_PARLI         NKYQITGDQDIYRNIPIWGNNSYFYNHTQNKNFELGNKSIIVEHVHEVVVENPV-GIISQ 1269
TR|Q964G1|Q964G1_PSEDP         NKYRLTGEQDIYSQIPIWGNNSYFYDHTLNKNFELGNHSVIVEHVRTVVVERPIPGIVSQ 1273
TR|Q7Z1Y6|Q7Z1Y6_HEMPU         NKYRLTGEQDIYRQIPIWGNNSYFYDHTLNKNFELGNHSIIVENVQTVVVDRPIPGISSQ 1270
TR|Q3YL94|Q3YL94_MESNU         NKYSLRGEQDIYNQIPIWGNNSYFYDHTLSKNFELGNHSIIVEHVQTVVGERPISGVMSQ 1273
TR|A0A1B4Z330|A0A1B4Z330_MESNU NKYSLRGEQDIYNQIPIWGNNPYFYDHTLSKNFELGNHSIIVEHVQTVVGERPISGVMSQ 1273
SP|P19615|MYP_STRPU            NKYVLTGEQDIYRQIPIWGNNSYFYDHTLNKNFELGNHSIIVEHVQTVVVERPSPGILSQ 1281
TR|Q8WQY0|Q8WQY0_TRIGR         NQYHLTGEQDVYRQIPIWGNNSFFYNHSLNKNFELGNHSVLVEHVQTVVVENPV-GVISQ 1266
TR|Q8T3T1|Q8T3T1_LYTVA         ------------------------------------------------------------
TR|C4TQH8|C4TQH8_STIJA         KNHIITE--NIETMMKLWGNEEFWTNHTLNSNFEIGVVNVTAMKNETEK----------- 1288
TR|C4TQH7|C4TQH7_STIJA         LNHTITE--HFETLMPLWGNKAFWMNHTLNSNFMIGVPKNVTRKDTTTL----------- 1309

TR|Q6WQT5|Q6WQT5_PARLI         INTDVDPEIAVQMDTAVINKTCEAVWTGQSWLPERFSDSKTTGSCVVPEYGANAKSRLSR 1329
TR|Q964G1|Q964G1_PSEDP         VNPEVDPEVQVQMDTVNISKTCESVWNGQSWLPERFPDSKISGSCVAPEYGVNAKSRVDR 1333
TR|Q7Z1Y6|Q7Z1Y6_HEMPU         VNLEVDPEVQVQMDSASITKTCETVWNGQSWLPERFQGYKTSGSCVIPEYGANAKSRVDR 1330
TR|Q3YL94|Q3YL94_MESNU         RIPEVDSEVQVQMDTVSITKTCESVWNGQSWLPERFPGYKTSGSCAVPEYGVNAKSRVER 1333
TR|A0A1B4Z330|A0A1B4Z330_MESNU RIPEVDSEVQVQMDTVSITKTCESVWNGQSWLPERFPGYKTSGSCAVPEYGVNAKSRVER 1333
SP|P19615|MYP_STRPU            VNSEVDPEVQVQMDSASLTKTCETVWNGQSWLPERFQGYKTSGSCVVPETGANAKSRVDR 1341
TR|Q8WQY0|Q8WQY0_TRIGR         LNTEVDPEDRVLMDTANITKTCESVWTGQSWLPERFQNYKTTGSCVVPEYGRNLRSRVSR 1326
TR|Q8T3T1|Q8T3T1_LYTVA         ------------------------------------------------------------
TR|C4TQH8|C4TQH8_STIJA         ---------LGPLTVLKSVGSCEANWYGNGWNTEWFVNTSRP-VCLGTIPGLRRTLTAQR 1338
TR|C4TQH7|C4TQH7_STIJA         ---------LGDVN-VKPLDTCEANWYGFEWHKEWFPETNRP-VCLGTVNGLRRVLTSER 1358

TR|Q6WQT5|Q6WQT5_PARLI         FRE-TMLRRQRQRQE---- 1343
TR|Q964G1|Q964G1_PSEDP         FRE-MMRRKQQIVDHHH-- 1349
TR|Q7Z1Y6|Q7Z1Y6_HEMPU         FRQ-IMQRKHQMVDHHH-- 1346
TR|Q3YL94|Q3YL94_MESNU         FRQ-IMQRKQQLVDHHH-- 1349
TR|A0A1B4Z330|A0A1B4Z330_MESNU FRQ-IMQRKQQLVDHHH-- 1349
SP|P19615|MYP_STRPU            FRQ-IMQRKQQLVDHHH-- 1357
TR|Q8WQY0|Q8WQY0_TRIGR         FRE-IMQRQREQERENYWQ 1344
TR|Q8T3T1|Q8T3T1_LYTVA         -------------------
TR|C4TQH8|C4TQH8_STIJA         IQTKLMP------------ 1345
TR|C4TQH7|C4TQH7_STIJA         VQTKIMP------------ 1365
```

Supplementary Figure 2. Intrinsic fluorescence and far UV CD spectra of toposome collected from control and T0 animals. Intrinsic fluorescence spectra (panels a and b) were measured in 10 mM Tris-HCl buffer at pH = 7.8 and 20 °C at 0.05 mg mL^{-1}. CD spectra (panels c and d) were measured in 10 mM Tris-HCl buffer at pH = 7.8 and 10 °C at 0.1 mg mL^{-1}.

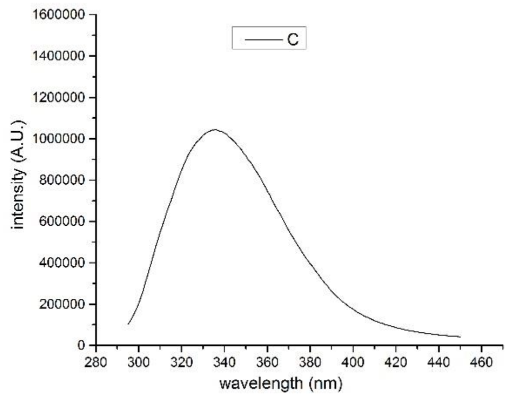

A

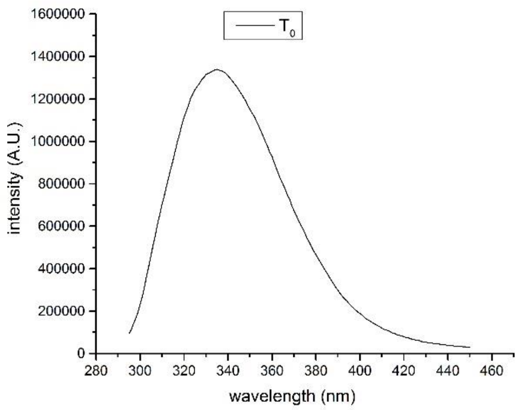

B

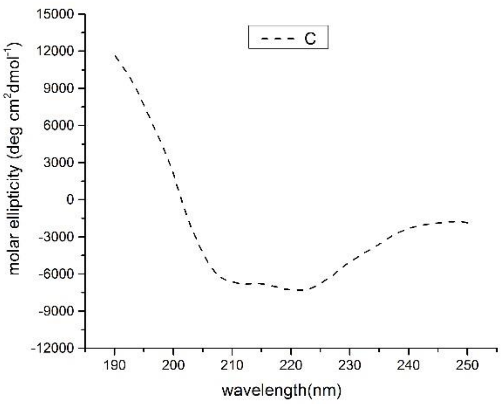

C

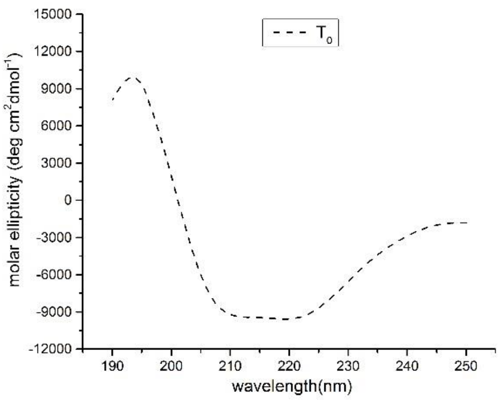

D

Supplementary Figure 3. Heat denaturation of toposome collected from control (panel A) and T0 animals (panel B), in the presence (red dotted lines) and in the absence of Ca^{2+} (black dotted lines), respectively, as followed by CD spectroscopy at 222 nm. Measurements were carried out in 10 mM Tris HCl buffer at pH 8.0, using a protein concentration of 0.2 mg mL^{-1}. The heating rate was 1 °C min^{-1}.

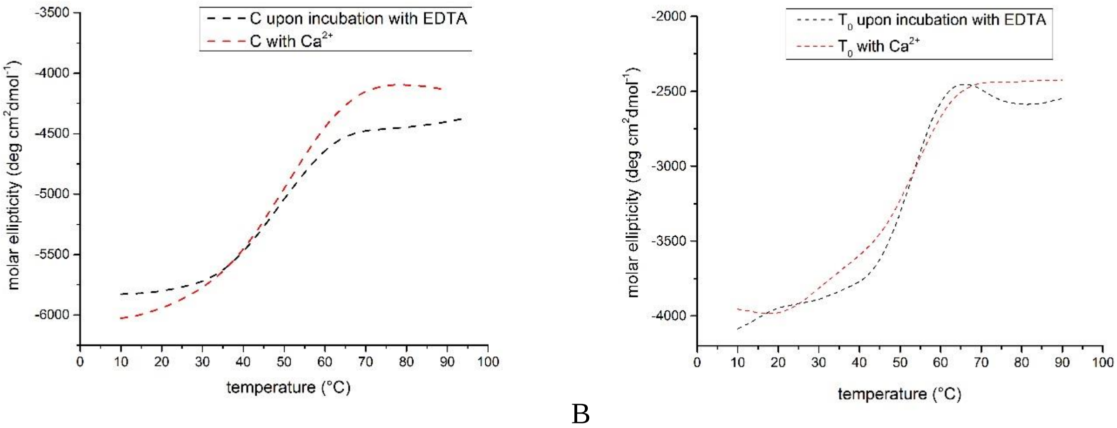

A B
