# Supplementary material for: Biotic and environmental stress induces nitration and changes in structure and function of the sea urchin major yolk protein toposome
Source: Sci Rep. 2018 Mar 15;8:4610. doi: 10.1038/s41598-018-22861-1 (PMC5854732; doi:10.1038/s41598-018-22861-1)
Supplement: Supplementary file 2 — Dataset 1, Dataset 2. [file 41598_2018_22861_MOESM2_ESM.docx]

**Biotic and environmental stress induces nitration and changes in structure and function of the sea urchin major yolk protein toposome**

Immacolata Castellano, Oriana Migliaccio, Giarita Ferraro, Elisa Maffioli, Daniela Marasco, Antonello Merlino, Adriana Zingone, Gabriella Tedeschi & Anna Palumbo

**Supplementary Table 1**. Nitrated peptides of toposome

The peptides refer to toposome in the control (C) collected prior to the bloom, at the bloom phase (T0), and after stabulation in sea water for 56 (T1) and 92 (T2) days. For each peptide, the table reports the sequence, the modification present, the value of X correlation (Xcorr), the charge, the m/z value in Da, the MH^+^ of the parent ion and the nitrated tyrosine.

**Supplementary Table 2**. Nitrated peptides in sea urchin embryos after metals treatment.

| **Cd 60kDa band** |  |  |  |
| --- | --- | --- | --- |
| Sequence | Modifications | MH+ [Da] | XCorr |
| _958_ SGVADMAFFDEQTLR _972_ |  | 1686.79082 | 5.21 |
| _440_ SGRPLNQNVIFLDESNTLK _458_ |  | 2145.14580 | 5.12 |
| _733_ NDPNTPMNVELLDDLVGVEGLSDLVK _758_ | M740(Oxidation) | 2812.41631 | 5.10 |
| _589_ YPDLLNVPEGQPK _601_ |  | 1469.76872 | 4.84 |
| _1099_ DQTGICYGETYTNIVK _1114_ | C1104(Carbamidomethyl) | 1861.87346 | 4.62 |
| _958_ SGVADMAFFDEQTLR _972_ | M964(Oxidation) | 1702.78044 | 4.74 |
| _779_ LGNAFPNFEALTPLSDK_795_ |  | 1833.94328 | 4.48 |
| _250_ TTCHAGINMPASFADPVCHLIK _271_ | C252(Carbamidomethyl); C267(Carbamidomethyl) | 2440.17514 | 4.61 |
| _25_ EGSCPPPPDAETQMSATR _42_ | C28(Carbamidomethyl); M38(Oxidation) | 1946.84038 | 4.42 |
| _889_ LSQTFVEPFDNVELR _903_ |  | 1793.92217 | 4.35 |
| _857_ TIPFLTDYKDEEITTVLK _874_ |  | 2126.14092 | 4.55 |
| _1290_ TCEAVWTGQSWLPER _1304_ | C1291(Carbamidomethyl) | 1819.85491 | 4.32 |
| _411_ MMQIYGNTDPTR _422_ | M411(Oxidation); M412(Oxidation) | 1458.64177 | 4.28 |
| _845_ ISTLEEIISHVK _856_ |  | 1368.78166 | 4.15 |
| _411_ MMQIYGNTDPTR _422_ |  | 1426.64409 | 4.01 |
| _648_ YFQATCIPEIEPETFR _663_ | C653(Carbamidomethyl) | 2000.95976 | 4.21 |
| _25_ EGSCPPPPDAETQMSATR _42_ | C28(Carbamidomethyl) | 1930.83098 | 3.90 |
| _1208_ ESNKYQITGDQDIYR _1223_ |  | 1829.86662 | 4.05 |
| _584_ SSLSKYPDLLNVPEGQPK _601_ |  | 1972.04607 | 3.87 |
| _411_ MMQIYGNTDPTR _422_ | M412(Oxidation) | 1442.64409 | 3.99 |
| _237_ LVNPNTFAELQDK _249_ |  | 1488.77544 | 3.79 |
| _1310_ TTGSCVVPEYGANAK _1324_ | C1314(Carbamidometh.) | 1553.72942 | 3.79 |
| _875_ HPAIMSYVEIYFPR _888_ |  | 1722.87566 | 3.63 |
| _920_ INNFIEMVK _928_ |  | 1107.59319 | 3.61 |
| _148_ MVNEFTYDVNMVPR _161_ | M141(Oxidation); M151(Oxidation) | 1746.79265 | 3.60 |
| _148_ MVNEFTYDVNMVPR _161_ |  | 1714.79326 | 3.95 |
| _937_ TCNSNLPLNFK _947_ | C2(Carbamidomethyl) | 1307.64409 | 3.50 |
| _980_ VGFTYNDLR _988_ |  | 1084.54497 | 3.51 |
| _664_ WDSDLLLGR _672_ |  | 1074.56572 | 3.46 |
| _410_ KMMQIYGNTDPTR _422_ | M412(Oxidation) | 1570.74504 | 3.33 |
| _504_ GIAENVPEVK _513_ |  | 1055.57402 | 3.31 |
| _638_ IGSVFESVNR _647_ |  | 1107.58159 | 3.39 |
| _910_ YTNPLWLSPK _919_ |  | 1218.65654 | 3.30 |
| _778_ LGNAFPNFEALTPLSDKIEMINK _801_ |  | 2562.34551 | 4.00 |
| _759_ GLQQTMGPEGR _769_ |  | 1173.57524 | 3.07 |
| _148_ MVNEFTYDVNMVPR _161_ | M158(Oxidation) | 1730.79973 | 3.67 |
| _1213_ YQITGDQDIYR _1223_ |  | 1371.65715 | 2.89 |
| _553_ EFLLDPLMSVHR _564_ |  | 1456.77239 | 3.36 |
| _1036_ ALMIAHHSPALPALFGEHTVMGK _1058_ |  | 2428.28244 | 2.78 |
| _410_ KMMQIYGNTDPTR _422_ | M411(Oxidation); M412(Oxidation) | 1586.73612 | 2.68 |
| _814_ DTPFGNLVQELFQGQLMVDIFGK _836_ |  | 2596.33445 | 2.64 |
| _759_ GLQQTMGPEGR _769_ | M764(Oxidation) | 1189.57317 | 2.59 |
| _542_ ACPVETFIAGK _552_ | C543(Carbamidomethyl) | 1192.60539 | 2.59 |
| _1164_ MADFQCDNGYGYLKPVVTAVACECMPCEER _1193_ | C1169(Carbamidomethyl) C1185(Carbamidomethyl);  C1187(Carbamidomethyl); M1188(Oxidation); C1191(Carbamidomethyl) | 3586.50876 | 2.50 |
| _866_ DEEITTVLK _874_ |  | 1047.55840 | 2.42 |
| _1091_ SSSYDWFK _1098_ |  | 1019.44646 | 2.33 |
| _1248_ SIIVEHVHEVVVENPVGIISQINTDVDPEIAVQMDTAVINK _1289_ |  | 4464.36977 | 2.31 |
| _920_ INNFIEMVK _928_ | M926(Oxidation) | 1123.58367 | 2.83 |
| _162_ KEWKCVQATCQEQCMFWIEQGWADIMTTR _190_ | C166(Carbamidomethyl); C171(Carbamidomethyl); C175(Carbamidomethyl); M176(Oxidation); W178(Nitro); M187(Oxidation); T88(Phospho) | 3877.61826 | 2.16 |
| _553_ EFLLDPLMSVHR _564_ | M560(Oxidation) | 1472.76555 | 2.40 |
| _673_ EMNWGFPTLNMYNFTGQEWMLWNTPATWNFLTYNRKVSK _711_ | M674(Oxidation); W676(Nitro); W700(Nitro); Y705(Nitro) | 4967.22866 | 2.10 |
| _602_ YIKDLWK _608_ |  | 965.54601 | 2.04 |
| _842_ SDKISTLEEIISHVKTIPFLTDYK _865_ | T857(Phospho); T62(Phospho); Y864(Phospho) | 3017.42612 | 2.03 |
| _842_ SDKISTLEEIISHVK _856_ |  | 1698.93584 | 2.02 |
| _1213_ YQITGDQDIYRNIPIWGNNSYFYNHTQNK _1241_ | T1216(Phospho); Y1222(Nitro); Y1235(Nitro); T1238(Phospho) | 3812.55942 | 2.01 |
| _305_ NGTYPLSLVSLCEDQQYEYSGIKGALK _331_ | Y308(Nitro); S314(Phospho); C316(Carbamidomethyl); Y321(Nitro) | 3203.39463 | 1.98 |
| _1194_ IEYNTSFTQDYMWSKESNKYQITGDQDIYR _1223_ | W1206(Nitro); S1207(Phospho); Y1213(Nitro) | 3893.64145 | 1.96 |
| _857_ TIPFLTDYK _865_ |  | 1097.59184 | 1.95 |
| _1091_ SSSYDWFKDQTGICYGETYTNIVK _1114_ | C1104(Carbamidomethyl) | 2862.26456 | 3.16 |
| _465_ AYAHHVYAAYNTCSKLTPKPR _485_ | Y466(Nitro); Y471(Nitro); Y474(Nitro); C477(Carbamidomethyl) | 2583.20157 | 1.92 |
| _134_ WCVSNQCQMTKCQRMVNEFTYDVNMVPR _161_ | C135(Carbamidomethyl); C140(Carbamidomethyl); T143(Phospho); C145(Carbamidomethyl); M138(Oxidation); Y21(Phospho); M25(Oxidation) | 3773.46311 | 1.91 |
| _930_ HQAEITKTCNSNLPLNFKGYEGSLR _954_ | T934(Phospho); C937(Carbamidomethyl); Y948(Phospho); S951(Phospho) | 3117.35556 | 1.90 |
| _191_ EGEVYTANTTFNLKPIAYETTINDEQPEVQILK _223_ |  | 3768.91623 | 1.89 |
| _608_ LKICSAGLRNFSAFSNPIGYLLANGTIPR _637_ | C611(Carbamidomethyl); S619(Phospho); S622(Phospho); Y627(Phospho) | 3390.58591 | 1.88 |
| _717_ QLLELK _722_ |  | 743.46660 | 1.85 |
| _411_ MMQIYGNTDPTRAFNIFDSSVYDCDTCKK _439_ | M411(Oxidation); M412(Oxidation); Y415(Nitro); Y432(Phospho); C434(Carbamidomethyl); C437(Carbamidomethyl) | 3634.47353 | 1.84 |
| _937_ TCNSNLPLNFKGYEGSLR _954_ | C938(Carbamidomethyl); Y949(Phospho) | 2149.96514 | 1.81 |
| _948_ GYEGSLRCLKSGVADMAFFDEQTLR _972_ | C955(Carbamidomethyl); S958(Phospho); M963(Oxidation) | 2946.31052 | 1.80 |
| _351_ IMSDEVER _358_ |  | 978.46105 | 2.38 |
| _638_ IGSVFESVNRYFQATCIPEIEPETFR _663_ | S641(Phospho); S645(Phospho); Y649(Nitro); C654(Carbamidomethyl) | 3294.43576 | 1.72 |
| _410_ KMMQIYGNTDPTRAFNIFDSSVYDCDTCK _438_ | Y415(Nitro); Y432(Phospho); C434(Carbamidomethyl); C437(Carbamidomethyl) | 3602.47922 | 1.68 |
| _1290_ TCEAVWTGQSWLPERFSDSK _1309_ | C1291(Carbamidomethyl); W1300(Nitro) | 2429.08261 | 1.67 |
| _465_ AYAHHVYAAYNTCSKLTPKPR _485_ | Y471(Phospho); C477(Carbamidomethyl); T481(Phospho) | 2608.16800 | 1.63 |
| _134_ WCVSNQCQMTKCQRMVNEFTYDVNMVPR _161_ | C135(Carbamidomethyl); S137(Phospho); C140(Carbamidomethyl); T143(Phospho); C145(Carbamidomethyl); Y154(Nitro) | 3786.46987 | 1.62 |
| _1059_ NFDMLIPIAPLNQSYQTYLGPKPLR _1083_ | M1062(Oxidation); Y1073(Nitro) | 2950.49814 | 1.61 |
| _1036_ ALMIAHHSPALPALFGEHTVMGK _1058_ | M1038(Oxidation); S1043(Phospho); T1054(Phospho) | 2604.18253 | 1.59 |
| _910_ YTNPLWLSPKINNFIEMVK _928_ | Y910(Nitro); T911(Phospho); W915(Nitro); S917(Phospho) | 2557.13432 | 1.58 |
| _406_ NLIKKMMQIYGNTDPTR _422_ | T421(Phospho) | 2102.99550 | 1.57 |
| _1224_ NIPIWGNNSYFYNHTQNKNFELGNK _1248_ | Y1235(Nitro); T1238(Phospho) | 3137.39975 | 1.56 |
| _648_ YFQATCIPEIEPETFRWDSDLLLGR _672_ | Y648(Nitro); T652(Phospho); C653(Carbamidomethyl); S666(Phospho) | 3261.36948 | 1.83 |
| _733_ NDPNTPMNVELLDDLVGVEGLSDLVK _758_ |  | 2796.41875 | 4.92 |
| _410_ KMMQIYGNTDPTR _422_ |  | 1554.74173 | 3.94 |
| _988_ LLCPNGQVVEIDVNLDIAK _1007_ | C555(Carbamidomethyl) | 2110.13921 | 3.73 |
| _553_ EFLLDPLMSVHRNNSVTLNHTYTR _576_ | S561(Phospho); T569(Phospho); T573(Phospho); T575(Phospho) | 3177.29331 | 2.95 |
| _1164_ MADFQCDNGYGYLKPVVTAVACECMPCEER _1193_ | C1169(Carbamidomethyl); C1185(Carbamidomethyl); C1187(Carbamidomethyl); C1190(Carbamidomethyl) | 3570.52585 | 2.81 |
| _778_ LGNAFPNFEALTPLSDKIEMINK _801_ | M797(Oxidation) | 2578.34214 | 2.62 |
| _337_ GQVTFVDQK _346_ |  | 1021.53447 | 2.59 |
| _465_ AYAHHVYAAYNTCSK _479_ | C477(Carbamidomethyl) | 1755.80033 | 2.49 |
| _1126_ DVTCVGTPR _1134_ | C1129(Carbamidomethyl) | 1004.48754 | 2.44 |
| _116_ YAPPQEQDRTPVTPNTIR _133_ |  | 2083.05913 | 2.09 |
| _1084_ SMEAIVKSSSYDWFKDQTGICYGETYTNIVK _1114_ | M1085(Oxidation); C2004(Carbamidomethyl); T2008(Phospho); Y2009(Phospho); T2010(Phospho) | 3876.55735 | 2.04 |
| _589_ YPDLLNVPEGQPKYIK _604_ | Y589(Nitro); Y602(Phospho) | 1998.94805 | 2.04 |
| _973_ DQDLLSRVGFTYNDLRLLCPNGQVVEIDVNLDIAK _1007_ | T983(Phospho); C591(Carbamidomethyl) | 4082.99082 | 1.99 |
| _502_ FKGIAENVPEVKNVAWGCILANSSMECMQAVHNNTADLYK _541_ | W517(Nitro); C519(Carbamidomethyl); C528(Carbamidomethyl); Y540(Nitro) | 4599.14956 | 1.98 |
| _305_ NGTYPLSLVSLCEDQQYEYSGIK _327_ | T307(Phospho); Y308(Nitro); S311(Phospho); C316(Carbamidomethyl); Y321(Nitro) | 2914.15995 | 1.96 |
| _1_ MRVAILLCLVASAVAAPSSMWGVREGSCPPPPDAETQMSATR _42_ | C8(Carbamidomethyl); M20(Oxidation); C28(Carbamidomethyl); M38(Oxidation); T41(Phospho) | 4582.11098 | 1.89 |
| _465_ AYAHHVYAAYNTCSKLTPKPRAK _487_ | Y466(Phospho); Y471(Phospho); Y474(Nitro); T476(Phospho); C477(Carbamidomethyl) | 2932.22751 | 1.74 |
| _1224_ NIPIWGNNSYFYNHTQNK _1241_ | Y1233(Phospho); Y1235(Nitro) | 2334.97226 | 1.67 |
| _410_ KMMQIYGNTDPTRAFNIFDSSVYDCDTCK _438_ | M409(Oxidation); M410(Oxidation); Y413(Nitro); C432(Carbamidomethyl); T434(Phospho); C435(Carbamidomethyl) | 3634.47133 | 1.59 |
| _875_ HPAIMSYVEIYFPRLSQTFVEPFDNVELR _903_ | T892(Phospho) | 3577.68894 | 1.55 |
| _1305_ FSDSKTTGSCVVPEYGANAKSR _1326_ | C1314(Carbamidomethyl); Y1319(Nitro); S1325(Phospho) | 2486.06852 | 1.54 |
| _1213_ YQITGDQDIYRNIPIWGNNSYFYNHTQNK _1241_ | Y1222(Nitro); Y1233(Nitro); T1238(Phospho) | 3732.58554 | 1.52 |
| _947_ GYEGSLRCLKSGVADMAFFDEQTLR _972_ | Y948(Phospho); C954(Carbamidomethyl); T969(Phospho) | 3010.29091 | 1.51 |
| _875_ HPAIMSYVEIYFPRLSQTFVEPFDNVELR _903_ | S880(Phospho); Y881(Phospho); Y885(Phospho) | 3737.62754 | 1.50 |
| **Mn 60 kDa band** |  |  |  |
| Sequence | Modifications | MH+ [Da] | XCorr |
| _1099_ DQTGICYGETYTNIVK _1114_ | C6(Carbamidomethyl) | 1861.86943 | 4.87 |
| _958_ SGVADMAFFDEQTLR _972_ | M6(Oxidation) | 1702.78459 | 4.43 |
| _889_ LSQTFVEPFDNVELR _903_ |  | 1793.92241 | 4.30 |
| _1290_ TCEAVWTGQSWLPER _1304_ | C1291(Carbamidomethyl) | 1819.85747 | 4.60 |
| _411_ MMQIYGNTDPTR _422_ | M411(Oxidation); M412(Oxidation) | 1458.64238 | 4.16 |
| _779_ LGNAFPNFEALTPLSDK _795_ |  | 1833.95183 | 4.12 |
| _958_ SGVADMAFFDEQTLR _972_ |  | 1686.79241 | 4.31 |
| _411_ MMQIYGNTDPTR _422_ |  | 1426.65605 | 3.99 |
| _410_ KMMQIYGNTDPTR _422_ | M412(Oxidation) | 1570.74639 | 1.10 |
| _423_ AFNIFDSSVYDCDTCK _438_ | C434(Carbamidomethyl); C437(Carbamidomethyl) | 1941.81536 |  |
| _937_ TCNSNLPLNFK _947_ | C938(Carbamidomethyl) | 1307.64910 | 3.59 |
| _237_ LVNPNTFAELQDK _249_ |  | 1488.78386 | 3.49 |
| _504_ GIAENVPEVK _513_ |  | 1055.57475 | 3.49 |
| _411_ MMQIYGNTDPTR _422_ | M411(Oxidation) | 1442.65056 | 3.47 |
| _920_ INNFIEMVK _928_ |  | 1107.59184 | 3.38 |
| _1209_ ESNKYQITGDQDIYR _1023_ |  | 1829.87029 | 3.32 |
| _910_ YTNPLWLSPK _919_ |  | 1218.65569 | 3.33 |
| _980_ VGFTYNDLR _988_ |  | 1084.54387 | 3.29 |
| _440_ SGRPLNQNVIFLDESNTLK _458_ |  | 2145.14096 | 3.12 |
| _589_ YPDLLNVPEGQPK _601_ |  | 1469.77312 | 3.85 |
| _148_ MVNEFTYDVNMVPR _161_ | M148(Oxidation) | 1730.80376 | 3.15 |
| _664_ WDSDLLLGR _672_ |  | 1074.56243 | 3.29 |
| _1213_ YQITGDQDIYR _1223_ |  | 1371.66203 | 2.92 |
| _920_ INNFIEMVK _928_ | M926(Oxidation) | 1123.58647 | 2.30 |
| _638_ IGSVFESVNR _647_ |  | 1107.58232 | 2.10 |
| _542_ ACPVETFIAGK _552_ | C543(Carbamidomethyl) | 1192.60930 | 2.79 |
| _410_ KMMQIYGNTDPTR _422_ | M411(Oxidation); M412(Oxidation) | 1586.74472 | 1.10 |
| _410_ KMMQIYGNTDPTR _422_ | S440(Phospho); S54(Phospho) | 2305.06913 | 1.10 |
| _866_ DEEITTVLK _874_ |  | 1047.56011 | 2.49 |
| _857_ TIPFLTDYKDEEITTVLK _874_ |  | 2126.14432 | 0.94 |
| _1091_ SSSYDWFK _1098_ |  | 1019.45220 | 2.13 |
| _134_ WCVSNQCQMTKCQRMVNEFTYDVNMVPR _161_ | C135(Carbamidomethyl); C140(Carbamidomethyl); T143(Phospho); C145(Carbamidomethyl); T153(Phospho); Y154(Nitro) | 3786.46621 | 2.09 |
| _1126_ DVTCVGTPR _1134_ | C129(Carbamidomethyl) | 1004.48815 | 2.83 |
| _1224_ NIPIWGNNSYFYNHTQNK _1241_ | Y1233(Phospho); T1238(Phospho) | 2369.95053 | 1.98 |
| _191_ EGEVYTANTTFNLKPIAYETTINDEQPEVQILK _223_ | Y195(Phospho); T196(Phospho); Y208(Phospho) | 4008.81113 | 1.93 |
| _1194_ IEYNTSFTQDYMWSKESNKYQITGDQDIYR _1223_ | Y1196(Phospho); Y1213(Nitro); Y1222(Nitro) | 3893.62197 | 2.18 |
| _602_ YIKDLWKLK _610_ | Y602(Phospho); W607(Nitro) | 1331.67180 | 1.79 |
| _1091_ SSSYDWFKDQTGICYGETYTNIVK _1114_ | T1101(Phospho); C1104(Carbamidomethyl); Y1105(Phospho) | 3022.22719 | 1.70 |
| _459_ IIDDSKAYAHHVYAAYNTCSK _479_ | Y473(Nitro); T475(Phospho); C476(Carbamidomethyl); S477(Phospho) | 2632.04546 | 1.62 |
| _1143_ FGAKQYK _1149_ | Y1148(Nitro) | 886.44872 | 1.60 |
| _1213_ YQITGDQDIYRNIPIWGNNSYFYNHTQNK _1241_ | T1216(Phospho); Y1222(Nitro) | 3687.65805 | 1.59 |
| _1224_ NIPIWGNNSYFYNHTQNKNFELGNK _1248_ | W1228(Nitro); Y1235(Phospho); T1238(Phospho) | 3217.33335 | 1.55 |
| _1147_ QYKMIKMCSRPSK _1159_ | Y1148(Nitro); C1154(Carbamidomethyl); S1158(Phospho) | 1781.80718 | 1.55 |
| _125_ TPVTPNTIRWCVSNQCQMTK _144_ | T125(Phospho); C135(Carbamidomethyl); C140(Carbamidomethyl); T143(Phospho) | 2581.08927 | 1.54 |
| _272_ EGVIPVTGNYVESFSDFVQESCIPGVLNKTYNK _304_ | T278(Phospho); S284(Phospho); S292(Phospho); C293(Carbamidomethyl); Y302(Phospho) | 4010.64638 | 1.53 |
| _1305_ FSDSKTTGSCVVPEYGANAKSR _1326_ | T1311(Phospho); C1315(Carbamidomethyl); Y1320(Nitro) | 2486.07071 | 1.53 |
| _1_ MRVAILLCLVASAVAAPSSMWGVR _24_ | C8(Carbamidomethyl); S12(Phospho); S18(Phospho); S19(Phospho) | 2798.30326 | 1.52 |
| _589_ YPDLLNVPEGQPKYIK _604_ | Y589(Nitro); Y602(Phospho) | 1998.96379 | 1.51 |
| _759_ GLQQTMGPEGR _769_ |  | 1173.57695 | 3.34 |
| _465_ AYAHHVYAAYNTCSK _479_ | C477(Carbamidomethyl) | 1755.80143 | 2.30 |
| _1059_ NFDMLIPIAPLNQSYQTYLGPKPLR _1083_ | M1061(Oxidation); S1071(Phospho); Y1075(Phospho) | 3065.48014 | 2.26 |
| _1084_ SMEAIVKSSSYDWFKDQTGICYGETYTNIVK _1114_ | S1093(Phospho); C1104(Carbamidomethyl); T1108(Phospho); T1110(Phospho) | 3860.61692 | 2.02 |
| _1224_ NIPIWGNNSYFYNHTQNKNFELGNK _1248_ | W1228(Nitro); Y1233(Nitro); Y1235(Nitro); T1238(Phospho) | 3227.34995 | 2.01 |
| _148_ MVNEFTYDVNMVPR _161_ | M148(Oxidation); M158(Oxidation) | 1746.79643 | 1.91 |
| _866_ DEEITTVLKHPAIMSYVEIYFPR _888_ | T870(Phospho); T871(Phospho); Y881(Nitro); Y885(Nitro) | 3001.30588 | 1.69 |
| _162_ KEWKCVQATCQEQCMFWIEQGWADIMTTR _190_ | C166(Carbamidomethyl); C171(Carbamidomethyl); C175(Carbamidomethyl); M176(Oxidation); W178(Nitro); M187(Oxidation); T189(Phospho) | 3877.63181 | 1.65 |
| _759_ GLQQTMGPEGR _769_ | M764(Oxidation) | 1189.56767 | 2.71 |
| _866_ DEEITTVLK _874_ | T870(Phospho); T871(Phospho) | 1207.49455 | 1.57 |
| _338_ GQVTFVDQK _346_ |  | 1021.53649 | 3.37 |
| _1084_ SMEAIVK _1090_ |  | 777.42284 | 2.70 |
| _973_ DQDLLSR _979_ |  | 846.43785 | 2.29 |
| _1310_ TTGSCVVPEYGANAK _1324_ | C1314(Carbamidomethyl) | 1553.73442 | 2.20 |
| _95_ SEDQIR _100_ |  | 747.36705 | 2.09 |
| _125_ TPVTPNTIR _133_ |  | 998.56725 | 1.97 |
| _1084_ SMEAIVK _1090_ | M1085(Oxidation) | 793.41228 | 1.91 |
| _351_ IMSDEVER _358_ | M352(Oxidation) | 994.45714 | 1.86 |
| _814_ DTPFGNLVQELFQGQLMVDIFGK _836_ |  | 2596.32584 | 4.61 |
| _116_ YAPPQEQDR 124 |  | 1103.51995 | 1.82 |
| _351_ IMSDEVER _358_ |  | 978.45995 | 1.74 |
| _796_ IEMINK _801_ |  | 747.40837 | 1.63 |
| _814_ DTPFGNLVQELFQGQLMVDIFGK _836_ | M30(Oxidation) | 2612.32889 | 2.83 |
| _584_ SSLSKYPDLLNVPEGQPKYIK _604_ | S587(Phospho); Y589(Phospho); Y602(Nitro) | 2581.17624 | 1.97 |
| _459_ IIDDSKAYAHHVYAAYNTCSKLTPKPR _485_ | Y465(Nitro); Y470(Nitro); T475(Phospho); C476(Carbamidomethyl); S477(Phospho) | 3369.48960 | 1.53 |
| **Mn 120 kDa band** |  |  |  |
| Sequence | Modifications | MH+ [Da] | Xcorr |
| _889_ LSQTFVEPFDNVELR _903_ |  | 1793.91838 | 4.53 |
| _1099_ DQTGICYGETYTNIVK _1114_ | C6(Carbamidomethyl) | 1861.88201 | 4.09 |
| _237_ LVNPNTFAELQDK _249_ |  | 1488.78386 | 3.99 |
| _589_ YPDLLNVPEGQPK _601_ |  | 1469.77349 | 4.09 |
| _980_ VGFTYNDLR _988_ |  | 1084.54997 | 3.59 |
| _759_ GLQQTMGPEGR _769_ |  | 1173.57219 | 3.41 |
| _779_LGNAFPNFEALTPLSDK_795_ |  | 1833.95159 | 3.41 |
| _338_ GQVTFVDQK _346_ |  | 1021.53649 | 3.25 |
| _1126_ DVTCVGTPR _1134_ | C4(Carbamidomethyl) | 1004.48845 | 3.03 |
| _1213_ YQITGDQDIYR _1223_ |  | 1371.66203 | 3.51 |
| _504_ GIAENVPEVK _513_ |  | 1055.58074 | 3.00 |
| _411_ MMQIYGNTDPTR _422_ | M1(Oxidation) | 1442.64324 | 3.26 |
| _920_ INNFIEMVK _928_ | M7(Oxidation) | 1123.59123 | 2.64 |
| _1310_TTGSCVVPEYGANAK_1324_ | C5(Carbamidomethyl) | 1553.73894 | 2.51 |
| _542_ACPVETFIAGK_552_ | C2(Carbamidomethyl) | 1192.61101 | 2.37 |
| _1091_SSSYDWFK_1098_ |  | 1019.45427 | 2.36 |
| _411_MMQIYGNTDPTR_422_ | M1(Oxidation); M2(Oxidation) | 1458.64189 | 2.84 |
| _910_YTNPLWLSPK_919_ |  | 1218.66106 | 2.30 |
| _1084_SMEAIVK_1090_ |  | 777.42235 | 2.48 |
| _759_GLQQTMGPEGR_769_ | M6(Oxidation) | 1189.57121 | 2.25 |
| _973_DQDLLSR_979_ |  | 846.43389 | 2.24 |
| _351_IMSDEVER _358_ | M2(Oxidation) | 994.45848 | 2.19 |
| _95_ SEDQIR_100_ |  | 747.36705 | 2.16 |
| _937_TCNSNLPLNFK_947_ | C2(Carbamidomethyl) | 1307.64690 | 2.01 |
| _125_TPVTPNTIR_133_ |  | 998.56725 | 1.95 |
| _116_YAPPQEQDR_124_ |  | 1103.51543 | 1.94 |
| _351_IMSDEVER_358_ |  | 978.45671 | 2.19 |
| _796_IEMINK_801_ | M3(Oxidation) | 763.40373 | 1.77 |
| _148_MVNEFTYDVNMVPR_161_ | M11(Oxidation) | 1730.80376 | 1.76 |
| _958_SGVADMAFFDEQTLR_972_ |  | 1686.78911 | 2.53 |
| _1084_SMEAIVK_1090_ | M2(Oxidation) | 793.41228 | 1.60 |
| _948_GYEGSLR_954_ |  | 781.38738 | 1.59 |
| _411_MMQIYGNTDPTR_422_ |  | 1426.65605 | 3.59 |
| _958_SGVADMAFFDEQTLR_972_ | M6(Oxidation) | 1702.78874 | 3.23 |
| _920_INNFIEMVK _928_ |  | 1107.59685 | 2.61 |
| _1290_TCEAVWTGQSWLPER_1304_ | C2(Carbamidomethyl) | 1819.85954 | 2.46 |
| _637_IGSVFESVNR_647_ |  | 1107.58867 | 2.44 |
| _866_DEEITTVLK_874_ |  | 1047.56255 | 1.69 |
| _814_ DTPFGNLVQELFQGQLMVDIFGK_836_ |  | 2596.32584 | 4.98 |
| _814_DTPFGNLVQELFQGQLMVDIFGK_836_ | M17(Oxidation) | 2612.32889 | 2.59 |
| _845_ISTLEEIISHVK_856_ | S2(Phospho) | 1448.74175 | 1.99 |
